# Sex-Dependent Molecular Mechanisms of Lipotoxic Injury in Brain Microvasculature: Implications for Dementia

**DOI:** 10.3390/ijms21218146

**Published:** 2020-10-31

**Authors:** Saivageethi Nuthikattu, Dragan Milenkovic, John C. Rutledge, Amparo C. Villablanca

**Affiliations:** 1 Division of Cardiovascular Medicine, University of California, Davis, CA 95616, USA; snuthikattu@ucdavis.edu (S.N.); dragan.milenkovic@inra.fr (D.M.); jcrutledge@ucdavis.edu (J.C.R.); 2 Université Clermont Auvergne, INRA, UNH, CRNH Auvergne, F-63000 Clermont-Ferrand, France

**Keywords:** brain microvasculature, cognitive function, western diet, sex difference, multi-genomics

## Abstract

Cardiovascular risk factors and biologic sex play a role in vascular dementia which is characterized by progressive reduction in cognitive function and memory. Yet, we lack understanding about the role sex plays in the molecular mechanisms whereby lipid stress contributes to cognitive decline. Five-week-old low-density lipoprotein deficient (LDL-R −/−) male and female mice and C57BL/6J wild types (WT) were fed a control or Western Diet for 8 weeks. Differential expression of protein coding and non-protein coding genes (DEG) were determined in laser captured hippocampal microvessels using genome-wide microarray, followed by bioinformatic analysis of gene networks, pathways, transcription factors and sex/gender-based analysis (SGBA). Cognitive function was assessed by Y-maze. Bioinformatic analysis revealed more DEGs in females (2412) compared to males (1972). Hierarchical clusters revealed distinctly different sex-specific gene expression profiles irrespective of diet and genotype. There were also fewer and different biologic responses in males compared to females, as well as different cellular pathways and gene networks (favoring greater neuroprotection in females), together with sex-specific transcription factors and non-protein coding RNAs. Hyperlipidemic stress also resulted in less severe cognitive dysfunction in females. This sex-specific pattern of differential hippocampal microvascular RNA expression might provide therapeutic targets for dementia in males and females.

## 1. Introduction

Alzheimer’s disease (AD) is the most widespread neurodegenerative disease [1], the most common form of dementia, and has no known cure [2]. Nearly 50 million people around the world are currently living with dementia [3]. In the United States, AD is the fourth leading cause of death for females [4] and seventh leading cause of death for males [5]. By 2050, the number of AD cases is projected to increase to 14 million Americans [6]. Among the 5.8 million Americans with AD, two thirds are women [7] and women have a later onset but more rapid cognitive decline after a diagnosis of dementia than men [8]. However, these differences do not appear to be solely explainable by the longer life expectancy of women when compared to men.

Vascular contributions to cognitive impairment and dementia (VCID) or vascular dementia (VaD) are becoming increasingly recognized as important because VaD is the second most common form of dementia and is characterized by continual reduction in cognitive function and deterioration of memory [9]. Indeed, nearly 50% of AD patients have VCID pathology [10,11]. Unlike AD however, males are at a greater risk of developing VaD than females [12], although the reasons remain unclear. Cerebral small vessel disease also known as microvascular disease, is the most common cause of VaD [13]. Interestingly, studies have shown that cardiovascular disease (CVD) and dementia (AD and VaD) share common risk factors such as hyperlipidemia, although the mechanistic link has not been clearly investigated [14]. Therefore, understanding the pathobiology of how high lipid stress contributes to vascular dysfunction that results in VaD and AD is clearly important. We have previously shown in Apolipoprotein (ApoE)-deficient male mice that the western diet (WD) leads to Activating transcription factor 3 (ATF3)-mediated pro-apoptotic, inflammatory, and oxidative stress related neurovascular inflammation in brain microvessels, shedding light on one operative molecular mechanism [15]. Interestingly, we did not find this to be the operative mechanism in female mice (unpublished observations). Thus, the molecular mechanism of lipid induced cerebrovascular injury in males and females remains largely unexplored in experimental models. 

Low density lipoprotein receptor (LDL-R) deficient mice are genetically well characterized [16] and widely used models in the study of atherosclerosis and dietary lipid stress since the LDL receptor is crucial for clearing ApoE-containing lipoproteins [17]. The absence of LDL receptors prolongs the circulation of very low density lipoprotein (VLDL) and LDL in the blood, making LDL-R deficient mice a particularly useful model for studying the relationship between lipid metabolism and inflammatory processes [17], and this is of relevance to understanding neurovascular inflammation and the vascular determinants of dementia. 

In our recent studies, we performed genome-wide transcriptome analysis of the microvasculature in the hippocampus region, a key memory center in the brain, of female [18] and male [19] low density lipoprotein receptor deficient (LDL-R −/−) mice fed the western diet (WD). We demonstrated for the first time that hyperlipidemic stress modulates differential expression of 7% and 5.7% of the hippocampal microvascular genome in females and males, respectively, including both protein coding and non-coding genes (microRNAs, small nucleolar RNAs and long non-coding RNAs). In females, differential gene expression was associated with differential regulation of a number of important cellular pathways such as toll-like receptor signaling pathway, VEGF signaling pathway, Adherens junction, Ras signaling pathway and transcription factors such as CREB1 (cAMP Responsive Element Binding Protein), ESR1 (estrogen receptor 1), and YY1 (Yin Yang 1) to name a few [18]. However, in males, differential gene expression involved cellular pathways such as MAPK signaling, oxidative phosphorylation, gap junction, cytokine-cytokine receptor interaction and transcription factors like ETS1 (ETS Proto-Oncogene 1), FOXP3 (forkhead box P3), and GABPalpha (GA Binding Protein Transcription Factor) [19]. We have also identified differentially expressed miRNA gene targets belonging to pathways such as cell adhesion molecules (CAMs) and chemokine signaling in females, and insulin signaling and Nf-kB signaling in males. 

According to the Alzheimer’s Disease Research Network, there is ‘a growing body of research that confirms that biological sex plays a role in disease risk, as well as presentation and progression of dementia, but we still do not understand much about the role sex plays in etiology and prognosis’ [20,21]. Therefore, there is a clear imperative to better define additional biologic processes (including molecular ones), and emphasize issues of sex and gender, to gain a better understanding that will help lead to therapies for men and for women. This cannot be accomplished without considering sex as a biologic variable and clearly understanding the molecular mechanisms of the disease at the transcriptional and post-transcriptional level in males and in females. However, sex is not enough, and there is a need for the additional step of sex/gender-based analysis [22,23,24].

Thus, it is necessary to define the process in the two sexes by sex-disaggregated data since we do not have an understanding of the different and unique biology underlying sex differences in the vascular contribution of dementia and how a major risk factor, like hyperlipidemia, exerts differential genetic control at the level of the brain microvasculature in the two sexes. Defining this for the entire mouse genome in males and in females under comparable and replicable study conditions is absolutely key to doing more precise and relevant health related research that attends to sex as a fundamental biologic variable in dementia.

Furthermore, comparing the differentially expressed genes, pathways, and transcriptional and post-transcriptional regulatory mechanisms identified in our previous studies in males and females is crucial for identifying potential sex-specific drug targets for lipid induced microvascular endothelial injury relevant to vascular dementia. Thus, our current work is a comparative analysis of the sex-specific mechanisms that may render vasculo-protection from hyperlipidemic stress in females compared to males. By using a sex/gender-based analysis (SGBA) approach we aim to determine how sex-linked biology contributes to the molecular mechanisms for the genomic effects of a high fat diet and experimental hyperlipidemia on brain microvascular endothelium.

## 2. Results

### 2.1. Sex Differences in Weight, Lipids, Glucose and Insulin

The dietary treatment resulted in female mice weighing less than male mice for all baseline and post diet comparisons (*p* < 0.05), Supplement Figure S1. The expected weight gain in the study mice were as follows: mean weight for male WT mice at baseline was 21g and increased by an average of 24% when fed the CD and 39% when fed the WD. Mean weight for female WT mice at baseline was 16.3 g and increased by an average of 16% when fed the CD and 42% when fed the WD. Mean weight of LDL-R −/− male mice at baseline was 17.25 g and increased by an average of 62% when fed the CD and 74% when fed the WD. Mean weight of LDL-R −/− female mice at baseline was 14 g and increased by an average of 36% when fed the CD and 68% when fed with WD.

WT male mice fed the WD had statistically significant higher total cholesterol, HDL and LDL levels than the corresponding females (Supplemental Table S1). Male WT and LDL-R −/− mice fed with WD had higher insulin levels than female mice (Supplemental Table S2). There were no statistically significant differences between males and females for any of the experimental groups. 

### 2.2. Cognitive Function

**Figure 1:**
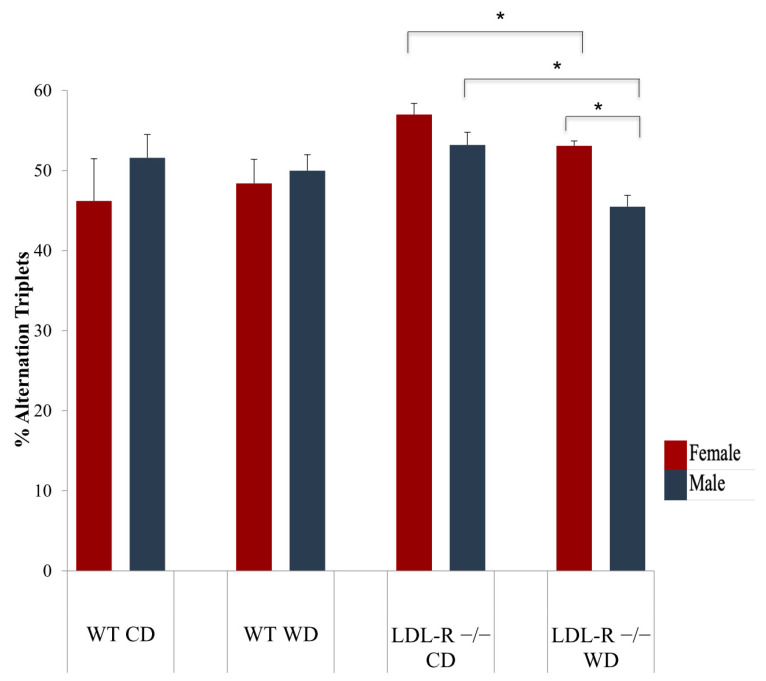
Sex and diet effect on cognitive performance. Cognitive performance was determined for female (red) and male (blue) mice in the Y-maze for all diet/genotype study groups: C57BL/6J (WT) mice fed western diet (WD), LDL-R −/− mice fed control diet (CD) and LDL-R −/− mice fed WD, when compared to WT mice fed CD. The percentage of alternation triplets (number of alternation triplets/ maximum number of alternation triplets) is shown (*n* = 5 female mice/experimental group were tested). Values are mean ± SEM alteration triplets. * *p* ≤ 0.05.

We used the y-maze to assess cognitive deficits in mice. The y-maze is a modification of the T- maze which evaluates memory and spatial learning (hippocampal) in rodents though quantification of alternating triplets (animal visits to all the zones of the y-maze consecutively, a measure of spatial cognition). Performance was assessed for differences in spontaneous alternation behavior (SAB) in the Y-maze related to the number of arm entries by the average of % alternation triplets (# alternating triplets/ total # triplets). As shown in Figure 1, WT female mice on the WD did not perform significantly different from WT female mice on the CD (48.4 ± 3.0 % alternation triplets vs 46.2% ± 5.3 % alternation triplets, respectively, *p* = ns). In contrast, female LDL-R −/− mice on the WD averaged statistically fewer alternation triplets (53.1% ± 0.6, when compared to female LDL-R −/− mice on the CD, 57% ± 1.4, respectively, *p* < 0.05). Thus, in females, cognitive performance assessed by SAB worsened with hyperlipidemic stress. We have previously reported impaired SAB in male LDL-R −/− mice fed the WD compared to CD [25]. Interestingly, with high dietary lipid stress (i.e., LDL-R −/− mice on the WD), SAB for females was statistically better than it was for males (53.1 % vs 45.5 % alternation triplets, respectively, *p* < 0.05), Figure 1. Thus, an analysis of differences in cognitive performance by spontaneous alteration behavior in the Y-maze identified a diet and sex interaction with females showing improved performance compared to males in the highest lipid stress condition (LDL-R −/− mice on the WD).

### 2.3. Principal Component Analysis

**Figure 2:**
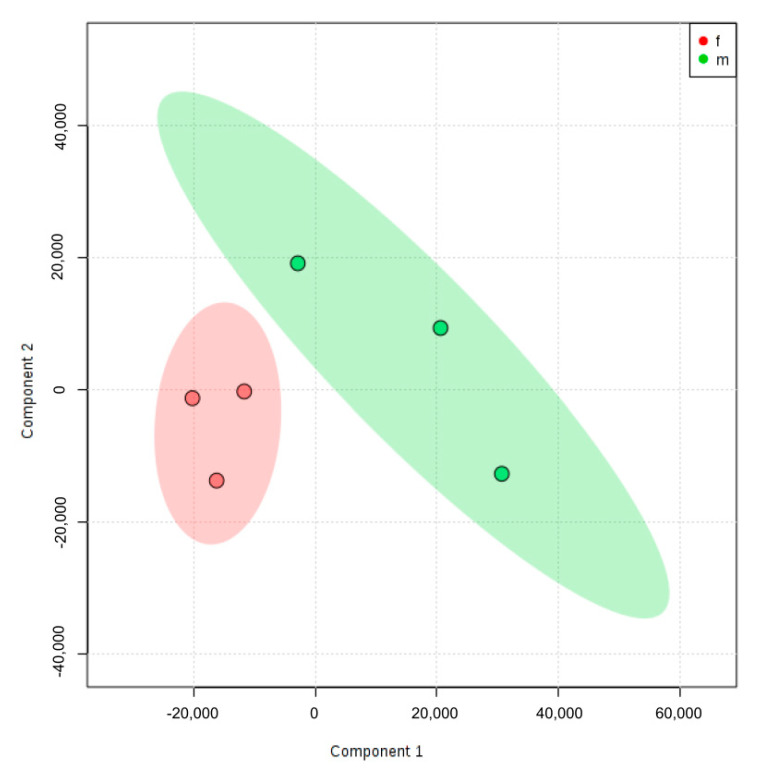
Principal Component Analysis (PCA) of differentially expressed genes in female and male mice hippocampal microvessels. PCA plot of the microarray data shows the trends of the expression profiles of the hippocampal microvasculature in female mice (f, red) and male mice (m, green) following the experimental diets and genotypes (*n* = 100 microvessels/mice/experimental group): C57BL/6J (WT) mice fed western diet (WD), LDL-R −/− mice fed control diet (CD), and LDL-R −/− mice fed WD, when compared to WT mice fed CD. The PCA plot captures the variance in a dataset in terms of principal components and displays the most significant of these on the *x* and *y* axes. The percentages of the total variation that are accounted for by the 1st and 2nd principal components are shown on the *x*- and *y*-axes labels.

Principal component analysis (PCA) is a tool to visualize genetic distance and relatedness between populations, the results of which are usually discussed in terms of component scores for each population studied. Importantly, PCA plot showed that the differentially expressed genes (DEG) for our experimental diet and genotype groups were distinctly different in females compared to males Figure 2. This suggests that the effect of the hyperlipidemic diet is fundamentally different at the genome level in male and female WT and LDL-R −/− mice.

### 2.4. Hierarchical Clustering

**Figure 3:**
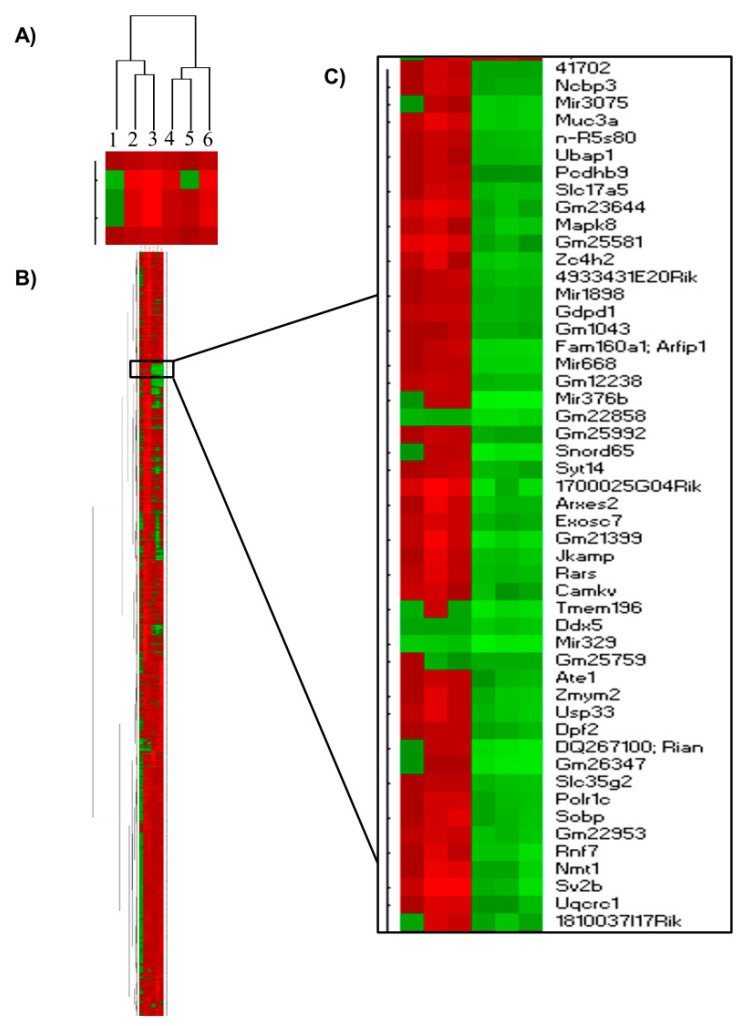
Hierarchical clustering of differentially expressed gene profiles. (**A**) Column comparison shows different clustering profile of male (columns 1–3) and female (columns 4–6) mice in the different diet/genotype groups (*n* = 100 microvessels/mice/experimental group): C57BL/6J (WT) mice fed western diet (WD) (column 1, 4), LDL-R −/− mice fed control diet (CD) (column 2,5), and LDL-R −/− mice fed WD (column 3,6). (**B**) Global hierarchical clustering. (**C**) Regions of cluster presenting opposite expression profiles between males (up regulated, red) and females (down regulated, green).

Hierarchical clustering groups similar data points together and then organizes the clusters into a hierarchy. Using hierarchical clustering we determined that there were clusters of differentially expressed genes (DEG) that were distinctly different in females compared to males with expression profiles that were at times in completely opposite directions, such as down regulated in females only and up regulated in male only, Figure 3. Some examples of DEGs showing different gene expression in females when compared to males included *Mapk8*, *Uqcrc1*, *Cdc26*, *Rnf7* and *Sv2b*. These DEG regulate 98 pathways including Alzheimer’s disease, ubiquitin mediated proteolysis, and extracellular matrix (ECM) receptor pathways, to name a few.

### 2.5. Differentially Expressed Protein Coding and Non-Coding RNAs

**Figure 4:**
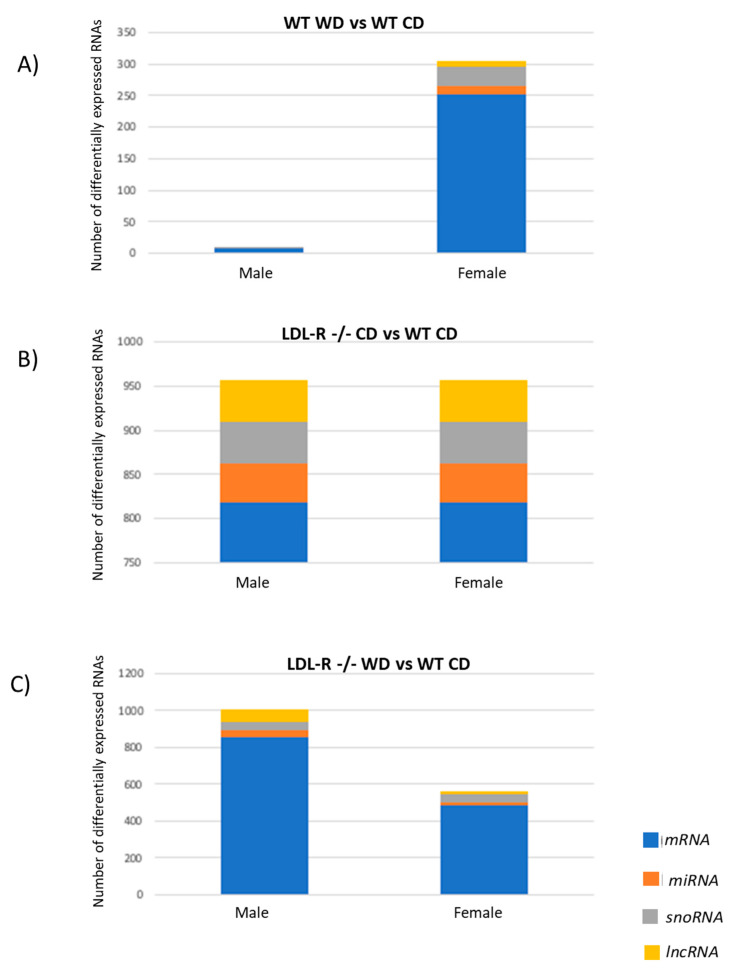
Sex differences in distribution of differentially expressed RNAs in hippocampal microvessels of male and female mice. Comparison of the number (*y*-axis) of differentially expressed mRNAs (blue), microRNAs=miRNAs (orange), small nucleolar RNA=snoRNAs (grey), and long non-coding RNA=lncRNAs (yellow) in hippocampal microvessels of male and female mice in the different diet/genotype groups (*n* = 100 microvessels/mice/experimental group). (**A**) C57BL/6J (WT) mice fed western diet (WD), (**B**) LDL-R −/− mice fed control diet (CD), and (**C**) LDL-R −/− mice fed WD, when compared to WT mice fed CD.

We found that the sex effect on differential RNA expression was dependent on the diet: in control WT mice on the WD, females had a substantially greater number of differentially expressed RNAs than males (observed previously) [18,19]), an equal number and proportion in LDL R−/− mice on CD, and a lower number in LDL R−/− mice on the WD. This sex pattern in differential RNA expression might be protective against lipid stress in females as we describe in the discussion. As shown in Figure 4, the total number of differentially expressed RNAs was greater in the LDL-R −/− groups compared to WT mice. In addition, the proportions were different between coding mRNA vs non-coding RNAs in that for WT on the WD and LDL R −/− mice on the WD most of the differentially expressed RNA was protein coding RNA, whereas in CD fed LDL R −/− mice differentially expressed RNAs were evenly distributed between protein coding and non-protein coding RNAs including micro RNA (miRNA), small nucleolar RNA (snoRNA) and long non coding RNA (lncRNA). Thus, when compared to WT mice on CD, the WD differentially regulated expression of mRNAs. In addition, LDL-R −/− mice on CD differentially regulated both protein-coding and non-protein coding RNAs equally.

To corroborate gene expression studies, a random sample of nine differentially expressed genes (DEG) for female mice and eleven DEG for male mice representative of each of the experimental genotype/diet groups, were tested by qRT-PCR and confirmed to have the same direction of change in gene expression (up- or down-regulation) as observed with the microarrays (Supplemental Figures S2 and S3).

### 2.6. Differentially Expressed Genes and Their Function

**Figure 5:**
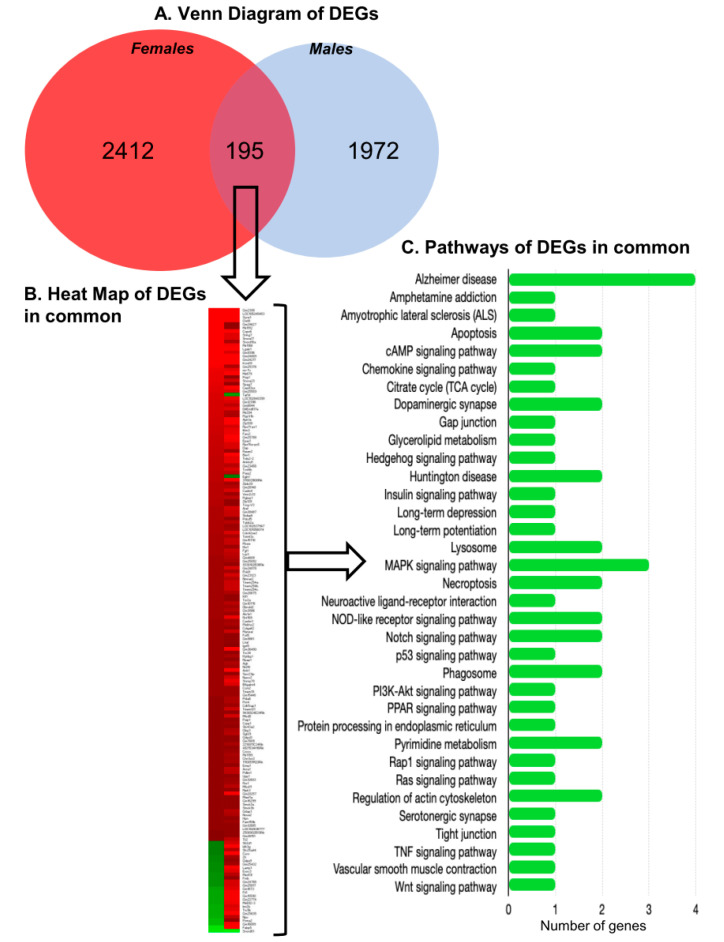
Sex differences in differentially expressed genes (DEG). (**A**) Venn diagram comparing the number of DEG genes in common between females (red) and males (blue) for all diet/genotype study conditions (*n* = 100 microvessels/mice/experimental group): C57BL/6J (WT) mice fed western diet (WD), LDL-R −/− mice fed control diet (CD) and LDL-R −/− mice fed WD, when compared to WT mice fed CD. (**B**) Heat map of DEGs. Note that 85% of the DEGs in common are in the same mode of expression in females and males; up regulated (red), down regulated (green). (**C**) Histogram of the pathways that the DEGs in common between males and females are involved in. See Supplemental Table S3 for a listing of all the genes in common.

In our system, there were a total of 2412 differentially expressed protein coding and non- coding genes in females and 1972 in males. Interestingly, and as shown in Figure 5, across all of the diet/genotype groups studied, only 195 of the differentially expressed genes (DEG) were in common between male and female mice. For the vast majority of common genes (85%), the direction of change of differential expression in males and females was the same (up regulation). Fifteen percent of the DEGs showed opposite expression patterns in males and females, with 25 genes down regulated in females and up regulated in males such as *Cycs*, *Npy*, *Fabp5*, *Exoc3*, *Lamp1* that regulate pathways such as apoptosis, tight junction, adipocytokine signaling pathway, stress induction of HSP regulation, phagosome, and also Alzheimer’s disease. In contrast, only two genes were up regulated in females and down regulated in males, *Egln1* (involved in hypoxia inducible factor 1) and transcription factor *Taf1d* (involved in RNA polymerase I-dependent transcription). 

The DEGs in common regulate a total of 35 pathways, each via a relatively small number of common genes (1–2 genes/pathway). The pathways regulated by the DEGs in common between males and females include regulation of apoptosis, gap junction, lysosomes, actin cytoskeleton, and tight junction. They also included a number of important signaling pathways: chemokine, cAMP, hedgehog, NOD-like receptor, notch, p53, Rap 1, Ras, and Wnt. A listing of all of the DEGs in common is provided in Table S3 in the Supplemental data.

### 2.7. Cellular Pathways

**Figure 6:**
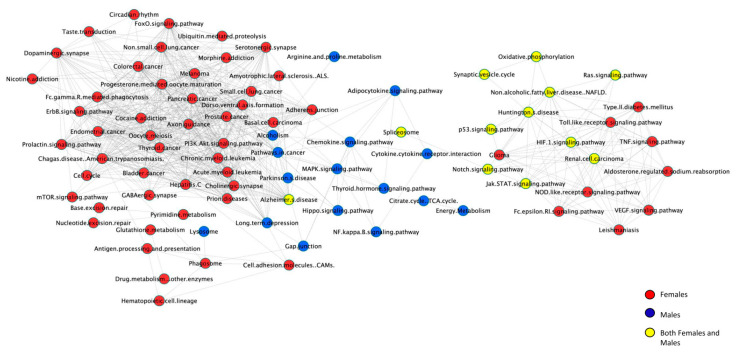
Sex differences in differentially expressed cellular pathways. Cellular pathways of differentially expressed protein-coding genes in hippocampal microvessels from female (red) and male (blue) mice for all diet/genotype study conditions (*n* = 100 microvessels/mice/experimental group): C57BL/6J (WT) mice fed western diet (WD), LDL-R −/− mice fed control diet (CD) and LDL-R −/− mice fed WD, when compared to WT mice fed CD. Pathways in common to both males and females are in yellow. Pathways were identified using KEGG and MetaCore. Pathway-connections network was built in Cytoscape.

In order to better understand functions in common or specific to males and females, we generated 2 separate histograms (one for males and one for females) to be able to analyze by sex and diet at the pathway level, Figure 6. This analysis also allowed us to generate a listing of pathways unique to females, unique to males, and common to both. We found that distinctly different cellular pathways are activated in males versus females. Male and female DEG together were involved in a total of 105 cellular pathways. Among these, 59 pathways were unique to females, 31 were unique to males, and only 15 pathways were in common for males and females. This is not surprising since, when compared to WT mice on the CD, there were more DEG, and thus there would be expected to be more pathways, in female vs male WT mice on the WD. Similarly, there were more DEG, and thus also more pathways, in LDL-R −/− mice on the CD and the WD, when compared to WT mice on the CD. The pathways with the greatest number of DEGs in females were the ubiquitin-mediated proteolysis and ribosome pathways. The pathways with the greatest number of DEGs in males were chemokine signaling, followed by Alzheimer’s disease, cytokine-cytokine receptor interaction, Huntington’s disease, and splicesome. Additional pathways unique to females and relevant to our work were: Adherens junction, Cell adhesion molecules, ErbB signaling, FoxO signaling, PI3K-Akt signaling, PPAR signaling, TNF signaling, Toll-like receptor signaling, Type II diabetes mellitus, Ubiquitin mediated proteolysis, and VEGF signaling. In contrast, pathways of particular interest to our work and unique to males were: Adipocytokine signaling, Chemokine signaling, Cytokine-cytokine receptor interaction, Gap junction, Hippo signaling, MAPK signaling, NF-κ B signaling, and Regulation of actin cytoskeleton. This suggests that in response to lipid stress, distinctly different cellular pathways are engaged in the hippocampal microvessels of males and females.

### 2.8. Pathway Networks

**Figure 7:**
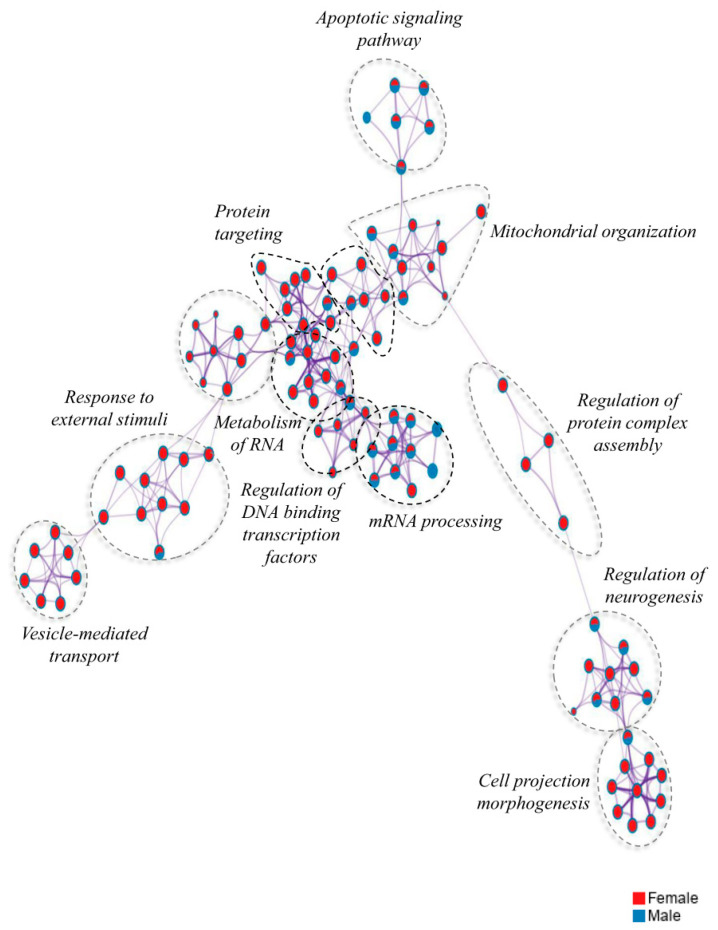
Functional networks of cellular pathways of differentially expressed protein-coding genes in hippocampal microvessels of male and female mice. ClueGo tool on Cytoscape was used to identify cellular pathways from the total number of differentially expressed genes in female vs the total number of differentially expressed genes in male hippocampal microvessels for all diet/genotype study conditions (*n* = 100 microvessels/mice/experimental group): C57BL/6J (WT) mice fed western diet (WD), LDL-R −/− mice fed control diet (CD), and LDL-R −/− mice fed WD, when compared to WT mice fed CD. The functional networks of cellular pathways for female mice (red circles), male mice (blue circles), and both female and male mice (red/blue circles) are shown.

Pathway networks were analyzed in order to look at the effect of male/female specific functions (as opposed the effect of diet), Figure 7. Functions that were mostly female specific included response to external stimuli, vesicle mediated transport, protein targeting, regulation of DNA binding transcription factors, metabolism of RNA, regulation of protein complex assembly, and cell projection morphogenesis. On the other hand, functions seen in both males and females included apoptotic signaling pathway, mRNA processing, mitochondrial organization, and regulation of neurogenesis. No pathway networks were seen to be more specific to males compared to females, which is not surprising given that the total number of DEGs was less in males than females.

### 2.9. Gene Ontology

**Figure 8:**
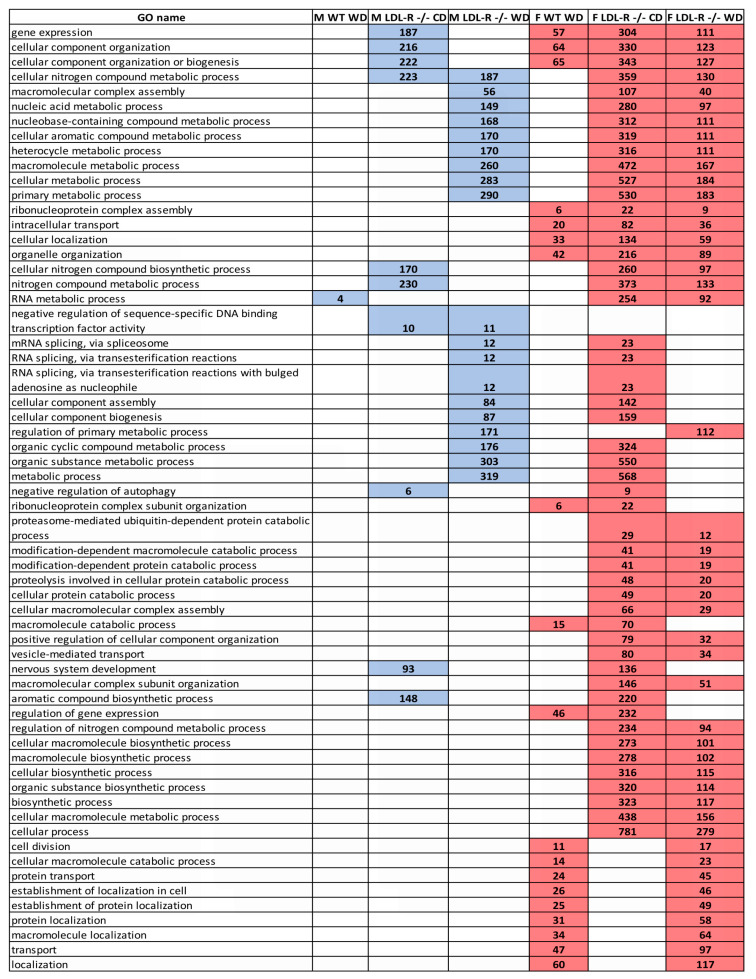
Sex differences in gene ontology of differentially expressed protein coding genes in hippocampal microvessels. David bioinformatics database was used to identify Gene Ontology (GO) significant (*p* < 0.05) biological processes for the total number of differentially expressed genes (DEG) in hippocampal microvessels from female and male mice for the following diet/genotype groups (*n* = 100 microvessels/mice/experimental group) when compared to C57BL/6J (WT) mice fed control diet (CD): WT mice fed western diet (WD), LDL-R −/− mice fed CD, and LDL-R −/− mice fed WD. GO biological processes found in two or more conditions (for diet and sex) are shown in the different colored rectangle boxes for male (blue) and female (red) mice. The number of DEGs for each of the GO biological processes is also provided.

Gene ontology (GO) provides a system for hierarchically classifying genes (in our case DEGs) into terms organized in a graph structure (ontology) in order to generate functional gene profiles to better understand the molecular function, biologic processes, and cellular components to ultimately better define the role of the DEGs. In general, GO analysis revealed that at the functional level, male mice had a much more limited molecular genetic response to lipid stress, whereas with increasing lipid stress female mice were able to engage a greater number and variety of biologic processes, Figure 8. In addition, in control male mice the WD differentially regulated only one RNA metabolic processes, whereas in LDL-R −/− mice the WD regulated nineteen cellular processes (including those involved in cellular nitrogen, aromatic, heterocycle, and macromolecule metabolism), as well as multiple RNA splicing mechanisms and cellular component assembly.

In contrast, in female control mice on the WD there was regulation of nineteen cellular processes (including cellular component organization, biogenesis, intracellular transport, cellular localization, organelle organization), as well as cellular macromolecular metabolism and catabolism and overall gene expression regulation. In the LDL-R −/− females, the WD played a role in nearly every gene ontology listed in Figure 8 with the exception of a very few (such as those involved in cellular and protein localization). Thus, the DEGs corresponded to far fewer genes and biologic processes in males than in females. 

In addition, although there was some overlap in the type of biologic processes for males and females on each diet (Figure 8), in general, the biologic process responses in males and female were quite different from each other. In this regard, there was no overlap at all for WD fed WT male and female mice, 18% overlap for CD fed LDL R −/− mice, and 22% overlap in WD fed LDL R −/− mice. In addition, females showed more biologic process redundancy which was observed for 10 biologic processes including gene expression, cellular organization and biogenesis, macromolecular complex assembly, RNA splicing, and nucleic acid metabolic processes.

### 2.10. Transcription Factors

**Figure 9:**
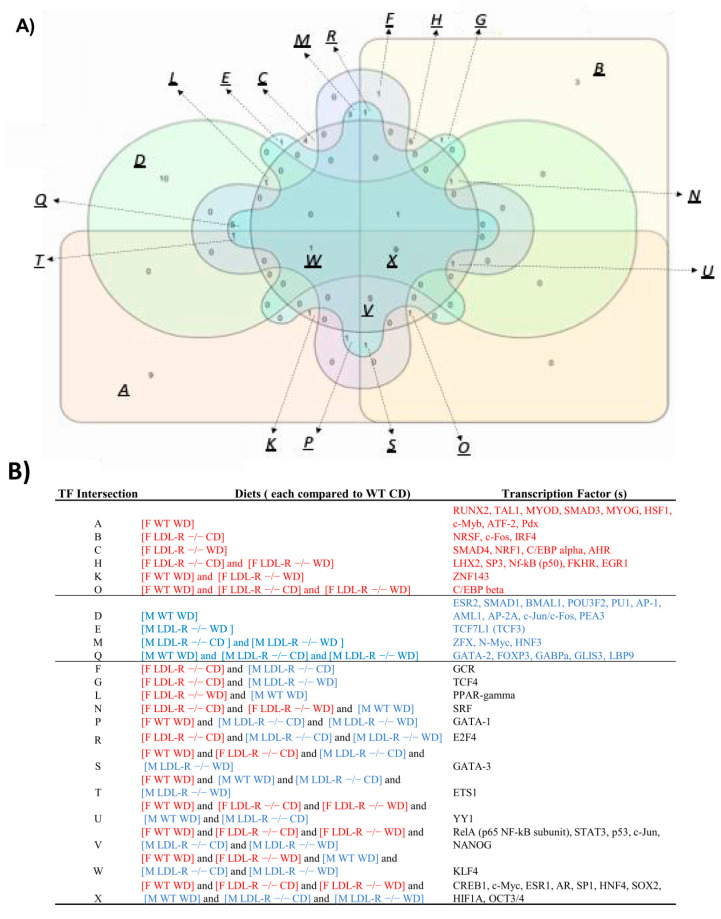
Venn diagram of the top 45 transcription factors affected by diet and genotype in hippocampal microvascular endothelium of female and male mice. Transcription factors potentially modulated by lipid injury in female and male mice were identified using MetaCore Transcription Regulation algorithm. (**A**) Venn diagram shows 9 transcription factors (TFs) (intersection X of Figure 9A,B) in common between female and male C57BL/6J (WT) mice fed western diet (WD), LDL-R −/− mice fed control diet (CD) and LDL-R −/− mice fed WD, when compared to WT mice fed CD (*n* = 100 microvessels/mice/experimental group). (**B**) Listing of TFs for each experimental diet comparing the TFs that are specific for females (red), specific for males (blue), and in common for both males and females (black).

In order to determine the sex specificity of transcription factors, if any, we compared identified transcription factors (TFs) between females and males as shown in Supplemental Figure S4. We identified 23 female specific TFs, 19 male specific TFs and 25 TFs in common. Furthermore, venn diagrams were constructed for TFs for females and males for all the diet and genotype groups, Figure 9. The expressed transcription factors differ in their identities in male and female mice. In females, RUNX2, TAL1, MYOD, SMAD3, MYOG, HSF1, c-Myb ATF-2, and Pdx were specific for the WD in WT mice; SMAD4, NRF1, C/EBP alpha, and AHR for the WD in LDL-R −/− mice; and ZNF143 for the WD in both. In contrast, in males, ESR2, SMAD1, BMAL1, POU3F2, PU1, AP-1, AML1, AP-2A, c-Jun/c-Fos, and PEA3 were specific for the WD in WT mice; and TCF7L1 (TCF3) for the WD in LDL-R −/− mice.

### 2.11. Non-Protein Coding RNAs

A heat map was performed for all non-protein coding RNAs. This demonstrated the following sex-specific patterns of non-protein coding RNA expression for snoRNAs, miRNAs, and lncRNAs: snoRNAs Gm23546, Gm12238, Gm23644, Gm25992, and Gm25635; MiRNAs Mir1898, and Mir668; and lncRNA Snhg14 and 4933431E29Rik were down-regulated in females but up- regulated in males. In contrast MiRNAs Mir290a and Mir337; sno RNA Gm25274 and Gm23613; and no lncRNAs were down-regulated in males and up-regulated in females. It is currently known that Snhg14 is a lncRNA that targets snoRNA, and Mir1898 targets 50 differentially expressed genes which are involved in pathways such as Ras signaling, Rap1 signaling, MAPK signaling, Ubiquitin mediated proteolysis, Tight junction and Alzheimer’s disease to name a few. There is currently no known function for the other non- protein coding RNAs mentioned and this remains an area of future research.

## 3. Discussion

In order to better understand the complexity and sex specificity of molecular regulation of the hippocampal brain microvasculature in response to the Western diet (WD), we characterized sex differences in the molecular mechanisms for the genomic effects of a high fat diet and experimental hyperlipidemia on brain microvascular endothelium of wild type and LDL-R-/- deficient mice (LR) by performing a global sex/gender transcriptomic analysis (SGTA) of laser-captured isolated microvessels from hippocampal regions of the brain. Our previous studies have shown that WD-induced lipotoxic injury significantly modifies the brain microvascular transcriptome in both male [19] and female [18] mice, but how the transcriptional modifications stratify by sex was not known. This is important to understand given: (a) the significance of brain microvessels in the pathogenesis of vascular dementia [13], (b) our recently published work [25] demonstrating that the WD results in increased blood brain barrier (BBB) permeability and cognitive impairment correlated to genomic modifications, and (c) the poorly understood gender differences in dementia between men and women. 

In this study, we identified a number of multiomic sex differences, as well as sex specificity, in the hippocampal microvascular molecular response to lipid stress between males and females. 

Below, we provide specific detailed examples of important sex differences in the complex molecular regulation of lipid stress following the WD on brain microvascular hippocampal endothelium that might explain the sex differences and relative neuroprotection in cognitive function observed in females in this study. To our knowledge, sex-specific effects of a high fat diet have not been previously reported (except for indices of the metabolic syndrome, specifically obesity related factors), [26].

### 3.1. Sex Differences in Hippocampal Microvascular Gene Expression and Hierarchical Clustering Following Lipotoxic Injury

Our study demonstrated the expected significant differences in weight, cholesterol and other lipid levels between control diet (CD) and the high fat WD fed to LR mice. The CD fed LR mice spontaneously demonstrated hyperlipidemia because of absence of the LDL receptor. In addition, differences in lipids between males and females in our model were consistent with those described previously [27,28,29]. We also demonstrated changes in serum glucose and insulin that were consistent with those previously published in similar experimental models, including the higher insulin levels observed in males [27,29,30].

Heretofore, it has been unknown whether and how a high fat diet accounts for sex differences in the transcriptome of brain hippocampal microvessels. The present study significantly extends our prior work and demonstrates for the first time that the WD and LR genotype significantly modulate sex differences in differential expression of approximately 5.7% of the hippocampal microvasculature genome of male mice, compared to 7% in female mice, for protein coding genes as well as non-coding RNA (miRNAs, snoRNAs and LncRNAs).

The PCA analysis also demonstrated for the first-time a clear separation between the sexes in response to diet in that the transcriptome of male samples in the diet and genotype groups clustered distinctly differently from female samples. Furthermore, analyses of the global expression profile of genes differentially expressed between males and females showed that the majority of genes were modulated in a dis-similar manner by lipotoxicity between males and females, upregulated in one and downregulated or unaffected in the other. In addition, we also identified clusters of differentially expressed genes that were downregulated by the WD in WT mice compared to CD, while their expression was upregulated in LR mice. These DEG included Serine/Threonine Kinase 3 (*Akt3*), Apolipoprotein A-I-Binding Protein (*Apoa1bp*), E3 Ubiquitin Protein Ligase 1 (*Siah1a*) and Ring Finger Protein 4 (*Rnf4*) which regulate cellular pathways such as MAPK signaling pathway [31], Notch signaling pathway [32], Ubiquitin mediated proteolysis [33] and NF-κB signaling pathway [34], respectively. Taken together, this study revealed complex genomic modifications of lipotoxicity in brain microvasculature involving different types of RNAs, revealing the importance of undertaking a multi-omic approach. Further analysis provides insight into the cellular functions of the differentially expressed genes. In future studies, it would be interesting to correlate these findings with proteomics.

### 3.2. Sex Differences in Differential Expression of Gene Ontology, Pathways, and Pathway Networks

Gene Ontology analysis of differentially regulated genes in our study showed a sex difference in the cellular and genetic response to lipid stress. Female mice exhibited a much greater molecular genetic response to lipid stress than did male mice. Among the many female-specific biological processes in response to lipid stress were ribonucleprotein complex, neurogenesis, and cellular localization and cellular macromolecules, all associated with neuroprotection. Ribonucleoprotein complex and neurogenesis are known to be enriched in hippocampal cells of mice exposed to acute stress and defend against oxidative stress [35], therefore preventing oxidative DNA damage which is a feature of Alzheimer’s disease (AD) and several other neurodegenerative diseases [36,37]. Cellular localization modulates synaptic function in the hippocampus [38], potentially contributing to neuronal plasticity. Lastly, cellular macromolecule metabolic process is associated with neuroprotective effects of anti-oxidant, B12 against H_2_O_2_-induced oxidative stress [39]. Thus, the greater number of biological processes differentially expressed following lipid stress in females compared to males correlate with differential expression of processes that may contribute to neuroprotection in females compared to males via antioxidant and neuroplasticity processes.

Sex-specific differentially expressed cellular pathways in female hippocampal microvessels following lipid stress included vascular endothelial growth factor (VEGF), PI3K-Akt, tumor necrosis factor (TNF), and peroxisome proliferator–activated receptor (PPAR) signaling. VEGF signaling is crucial for endothelial cell function related to angiogenesis [40]. Androgens enhance angiogenic events in male endothelial cells via VEGF-dependent mechanism [41]. However, in our study the VEGF signaling pathway was not activated in male mice following lipid stress. Moreover, recently it has been shown that decrease in VEGF expression is associated with cerebral small vessel disease in humans as observed by MRI [42]. The PI3K-Akt intercellular signaling transduction pathway contributes to angiogenesis through secretion of VEGF [43]. Cardio-protection has been shown to proceed via the PI3K/Akt1/2 pathway in males and the PI3K/Akt3 pathway in females [44]. However, the PI3K/Akt1/2 pathway was not activated in male mice following lipid stress in our study and may have resulted in lack of vasculo-protection in males. It has also been suggested that PI3K/Akt3 play a role in attenuation of cognitive deficits in rats with induced ischemic stroke [45]. The TNF signaling pathway is pleotropic in its effects and either promotes cell death, proliferation, or survival and differentiation [46]. Females cardiac progenitor cells treated with TNF-α receptor 2 have improved cell migration and proliferation compared to males [47]. This suggests that the TNF signaling pathway may play a vasculo-protective role following lipid injury in females by promoting endothelial cell proliferation and migration. Lastly, the PPAR-α signaling pathway plays an important role in cellular lipid utilization [48]. PPAR-α may also play a role in anti-oxidative and anti-inflammatory processes together with fatty acid transport, lipid metabolism and disturbances of mitochondria function in the brain and therefore regulate cognitive function, neurodegenerative and neurodevelopmental disorders [49]. PPAR gene deletion in male mice is lethal death due to massive cardiac lipid accumulation that can interestingly be reversed in males with estrogen treatment. In contrast PPAR gene deletion in females is lethal in only 25% of PPAR knockout mice [48]. This implies that lack of expression of PPAR signaling in hippocampal microvessels of male mice following lipid stress may render the microvascular endothelium of male mice more susceptible to lipid stress than in female mice. Taken together, the differentially expressed female-specific cellular pathways we identified in female hippocampal microvessels may contribute to relative neuroprotection in females following lipid stress through regulation of angiogenesis, endothelial cell proliferation and migration, and cellular lipid utilization.

Our bioinformatics analysis also revealed distinctly different pathway networks involved in males and females in response to lipid stress, with little overlap between males and females. Among the differentially expressed pathway networks specific to females was metabolism of RNA which we found to be differentially expressed in nearly eight female-specific pathways activated in response to lipid stress. Metabolism of RNA is of interest because disrupted RNA is a significant contributor to neurodegenerative diseases [50]. Furthermore, sequestration of RNA binding proteins by abnormal RNA compromises neuronal integrity, thus highlighting the susceptibility of neurons to deleterious changes in RNA expression [51]. Another differentially expressed pathway network specific to females was cell projection morphogenesis which is enriched in the hippocampus region of mice brain under acute stress and serves to counteract oxidative stress-induced DNA damage [35]. Thus, in females there appears to be specific hippocampal microvascular protection against disruption of RNA and oxidative stress-induced DNA damage following lipid stress and this may play a role in relative neuroprotection in females. 

### 3.3. Sex- Differences in Differential Regulation of Transcription Factors and their Inter- Connection to Signaling Pathways

In our study, functionally distinct and sex-specific transcription factors (TFs) were activated in male and female hippocampal microvessels following lipid stress. Female specific differentially expressed TFs included basic helix loop helix TAL1, GC binding SP3, RUNT related transcription factor 2 (RUNX2), and heat shock transcription factor (HSF1). TAL1 is linked to modulation of endothelial cell morphogenesis and migration [52]. SP3 mediates endothelial cell growth by increasing the activity of the VEGF receptor [53], important as VEGF signaling pathway promotes angiogenesis [40] and is activated in females following lipid injury as previously discussed. Similarly, RUNX2 induces VEGF expression and promotes endothelial cell proliferation [54]. Thus, both SP3 and RUNX2 TFs are functionally connected to the VEGF signaling pathway in females. Lastly, HSF1 is known to protect endothelial cells from endoplasmic reticulum stress-induced cell death [55]. This suggests that HSF1 may protect female hippocampal microvessels from apoptosis following lipid injury.

Male specific differentially expressed TFs included Activator protein 1 (AP1), Fork head box P3 (FOXP3), NF- κ B, and PU.1. AP-1 is composed of the c-Jun and c-Fos family of proteins [56]. c-Jun activates stress-induced apoptosis in hippocampus [57] and the c-Jun/AP-1 proteins can induce the release of neurodegenerative molecules that damage the nervous system [58]. In addition, AP-1 along with NF-κ B signaling pathway activates pro-inflammatory cytokines [59] that induce inflammation and neuronal apoptosis [60]. In mouse model of vascular dementia, during hypoxia, NF- κ B signaling pathway induces neuronal loss and contributes to cognitive dysfunction [61]. This implies that the transcription factor AP-1 and NF-κ B signaling pathway are activated in male hippocampal microvessels following lipid stress and may induce vascular damage through inflammation and apoptosis. FOXP3 is also pro-apoptotic [62] and plays a deleterious role in AD pathology by immunosuppression [63]. Increased levels of FOXP3 during disease progression has been observed in mouse model of AD [63] as well as AD patients [64]. This suggests that FOXP3 expression in male hippocampal microvessels following lipid stress may induce apoptosis and contribute to neurodegeneration. Lastly, the PU.1 transcription factor is activated by mitogen-activated protein kinase (MAPK) signaling pathway [65] and induces inflammatory responses in microglial cells of human brain with AD [66]. Thus in males, the AP1 transcription factor appears linked to NFK-B signaling whereas the PU.1 transcription factor is linked to the MAPK signaling pathway, suggesting that male specific activation of transcription factors AP1 and PU.1 following lipid stress may contribute to neuroinflammation and degeneration via the NFK-B and MAPK signaling pathways, respectively.

Taken together, our findings indicate that sex-specific differential expression of transcription factors unique to females contribute to signaling of cellular pathways that may improve endothelial cell health by promoting endothelial cell proliferation, VEGF signaling and angiogenesis, and protecting from stress-associated cell death. In contrast, differential expression of male specific transcription factors results in cellular signaling that may promote vascular injury by activation of stress induced apoptosis, neurodegeneration, and neuro-inflammation.

### 3.4. Sex- Differences in Differential Regulation of Non-Coding RNAs

Our microarray analysis also indicated marked sex differences and sex specificity in regulation of non-coding RNAs (ncRNAs), specifically microRNAs (miRNAs), small nucleolar RNAs (snoRNAs), and long non-coding RNAs (lncRNAs) with only 6% of the differentially expressed non coding RNAs in common between males and females (Supplement Figure S5). Here we provide a few examples of differentially regulated miRNAs, snoRNAs, and lncRNAs in males and females and the potential functional implications. The differential regulation of ncRNAs suggests that they may further contribute to neuroprotection and improved cognition in females, and to inflammation and cognitive impairment in males. 

In male mice following lipid stress we observed up-regulation of mir-34c. Increased expression of mir-34c in the hippocampus has been shown to impair memory and cognition in murine AD models and human AD patients [67,68]. In contrast, with lipid injury in female mice there was up-regulation of mir-539 which is known to play a neuroprotective role [69]. In AD mice, Mir-539 inhibits Aβ accumulation, a key process in the AD pathogenesis, decreases oxidative stress and apoptosis, and contributes to improved memory [69].

Female specific small-cajal body RNA 2 (SCARNA2), a snoRNA, was also up-regulated following lipid injury. SCARNA2 is known to improve ribosomal RNA modifications and generate ribosomes to produce proteins to cope with stress [70]. In contrast, lipid stress activated the expression of male specific snoRNA, RNU3A (SNORD3A), that is known to be associated with protein misfolding [71], a key contributor to brain degeneration [72]. SNORD3A also reduces resistance to oxidative stress induced lipid peroxidation [71,73] and hence may worsen stress induced pathological damage in neurodegenerative diseases such as AD [74].

Lastly, following lipid stress there was up-regulation of male specific lncRNA HOXA Cluster Antisense RNA 3 (HOXA-AS3)/. This is potentially of significance as HOXA-AS3 is known to induce endothelial inflammation via positive regulation of NF-κ B pathway [75]. In contrast, in females, specific differential down regulated expression of lncRNA small nucleolar RNA host gene 14 (SNHG14), known to increase neurological impairment and inflammatory response [76], may contribute to improved cognition in female mice. This is one of the first studies to show sex differences in non-coding RNAs in brain microvasculature following lipid stress.

## 4. Methods

### 4.1. Experimental Animals

5 week old low-density lipoprotein receptor deficient (LDL-R −/−; strain B6.129S7-Ldlr tm1Her/J, Jackson Laboratories, Bar Harbor, ME, USA) [16] and C57BL/6J wild type (WT; Jackson Laboratories, stock 000664) male and female mice were fed either a standard chow control diet (CD = Chow, Nestlé Purina PetCare Co., St. Louis, MO, USA) or a Western Diet (WD, catalog no. 88137, Harlan Laboratories, Madison, WI, USA) composed of 21% fat and 0.2% cholesterol (*w*/*w*) for 8 weeks at which point mice were 13 weeks of age and sacrificed. There were four experimental treatment groups randomly assigned to the diets for male and female mice: WT fed CD, WT fed WD, LDL-R −/− fed CD, and LDL-R fed WD; *n* = 7 mice/group. Animals were housed 2–3/cage in a temperature- and humidity-controlled environment with a 12 h light/dark cycle in the University of California, Davis Mouse Biology Program. Body weight was measure at baseline and at the completion of the dietary intervention period, and activity and food intake monitored daily by vivarium staff. Research was conducted in conformity with the Public Health Service Policy on Humane Care and Use of Laboratory Animals. The institutional review board of the University of California, Davis, the Institutional Animal Care and Use Committee (IACUC) approved this project protocol number 19750 on 7 February 2017.

### 4.2. Cognitive Testing (Y-Maze)

Female (*n* = 5 mice/group) that had completed the 4 experimental diet/genotype groups [WT fed CD, WT fed WD, LDL-R −/− fed CD, and LDL-R fed WD] were adapted to the testing room for 30 min, placed in the center of the *Y*-maze and tracked with an overhead camera for the extent of a 8 min trial. An elevated white plastic Y-maze with three 40 cm arms at 120° angles was utilized. Entry into each arm (arm 1–3) after entry to the center (arm 4), total distance traveled, latency to entry and frequency, and an alternation score was computed as the number of times the three arms were sequentially entered. The % alternation score is the number of alternations divided by maximum alteration triplets. Data are presented as means ± SEM. Similar method was used for testing of male mice (*n* = 8 per group) as previously described [25].

### 4.3. Blood Metabolic and Hormone Assays

Following completion of the dietary feeding period fasting lipid levels were measured in serum samples obtained at the time of sacrifice and stored at −80 °C until assayed. Total cholesterol (TC), high-density lipoprotein (HDL), and low-density lipoprotein (LDL) were measured using enzymatic assays from Fisher Diagnostics (Middleton, VA, USA), and precipitation separation from AbCam (Cambridge, MA, USA) adapted to a microplate format. Fasting glucose and insulin levels were also measured on serum samples. Glucose was measured using enzymatic assays from Fisher Diagnostics (Middleton, VA, USA), and insulin was determined by electrochemiluminescence from Meso Scale Discovery (Rockville, MD, USA) according to the manufacturer’s instructions. All assays were performed by the UC Davis Mouse Metabolic Phenotyping Center (MMPC) in triplicate, on non-pooled plasma samples. Data are presented as means ± SEM.

### 4.4. Isolation and Cryosection of Murine Brain Hippocampus

Following completion of the dietary feeding period, mice were anesthetized by intraperitoneal xylazine/ketamine and euthanized by exsanguination during the light phase of their light/dark cycle, then intravascularly perfused with DEPC-treated PBS. Intact brains were rapidly removed under RNAse free conditions, cut into regions including the temporal lobe segment, and embedded using HistoPrep Frozen Tissue Embedding Media (Fisher Scientific, Pittsburgh, PA, USA). To identify the hippocampus and hippocampal neurons, brain sections in the medial aspect of the temporal lobe were stained with hematoxylin and visualized with microscopy by a histopathology expert at UC Davis (Dennis Wilson). The hippocampus was then coronally cryosectioned (8 µm, Leica Frigocut 2800n Cryostat, Leica Biosystems, Buffalo Grove, IL, USA). Hippocampal cryosections were placed on charged RNA-free PEN Membrane Glass slides, treated with RNA*later*^®^-ICE (Life Technologies, Grand Island, NY, USA) to prevent RNA degradation, and stored at −80°C until use. When ready for use, cryosections from the hippocampal segments were submerged in nuclease-free water and dehydrated in desiccant.

### 4.5. Laser Capture Microdissection (LCM) of Hippocampal Microvessels

For analysis of gene transcriptome of hippocampal brain microvessels, endothelial microvessels (<20um) were first identified in the hippocampal brain cryosections by alkaline phosphatase staining utilizing 5-bromo-4-chloro-3-indolyl phosphate/nitro blue tetrazolium chloride (BCIP/NBT) substrate as previously described [77]. Laser capture microdissection (LCM) was then used to isolate the endothelium of the microvessels within the hippocampal sections by capture of the entire vessel wall under direct microscopic visualization using a Leica LMD6000 Laser Microdissection Microscope (Leica Microsystems, Wetzlar, Germany). Microvessels were not categorized by hippocampal region or subregion, although they primarily corresponded to endothelial enriched sections in hippocampus dorsal segments that would have included CA1 and CA3 regions. 

### 4.6. RNA Extraction from Laser Captured Brain Microvessels

Total RNA was extracted from the laser-captured hippocampal brain microvessels (300 microvessels/sample) from each of the four experimental animal groups using an Arcturus PicoPure™ RNA Isolation Kit (Thermo Fisher Scientific, Santa Clara, CA, USA) according to the manufacturer’s instructions. The quality of the RNA from the LCM-derived vessels was assessed by Nanodrop, and RNA integrity verified by qRT-PCR of control gene transcription (GAPDH). RNA quantification was performed according to Affymetrix RNA quantification kit with SYBR Green I and ROX™ Passive Reference Dye protocol (Affymetrix, Santa Clara, CA, USA). 

### 4.7. Microarray Hybridization and Transcriptome Analysis

For transcriptomics analysis we used Affymetrix GeneChip Mouse Gene 2.0 ST Array (~28,000 coding transcripts and ~7.000 non-coding transcripts, Affymetrix, Santa Clara, CA, USA). RNA (125 pg) was used to prepare cRNA and sscDNA using Affymetrix GeneChip^®^ WT Pico Kit (Affymetrix, Santa Clara, CA, USA). SscDNA (5.5 ug) was fragmented by uracil-DNA glycosylase (UDG) and apurinic/apyrimidinic endonuclease 1 (APE 1) and labeled by terminal deoxynucleotidyl transferase (TdT) using the DNA Labeling Reagent that is covalently linked to biotin. Fragmented and labelled sscDNA samples in triplicate were then submitted to the UC Davis Genome Center shared resource core for hybridization, staining, and scanning using Affymetrix WT array hybridization protocol following the manufacturer’s protocol. Hybridization of fragmented and labelled sscDNA samples was done using GeneChip™Hybridization Oven 645 (Thermo Fisher Scientific, Santa Clara, CA, USA), and samples then washed and stained using GeneChip™ Fluidics Station 450 (Thermo Fisher Scientific, Santa Clara, CA, USA). The arrays were scanned using GeneChip™ Scanner 3000 7G (Thermo Fisher Scientific, Santa Clara, CA, USA). Quality control of the microarrays was done using Affymetrix Expression Console software version 1.4.1, and data analysis performed using Affymetrix Transcriptome Analysis Console software version 3.1.0.5. 

### 4.8. qRT-PCR Analysis of Gene Expression in Murine Hippocampal Microvessels

To corroborate the microarray analysis results, we randomly selected 9 differentially expressed RNA transcripts for female mice and 11 for male mice and performed qRT-PCR as previously reported [18,19]. For these experiments, RNA (75 ng) from the laser-captured brain microvessels (*n* = 7 mice/group) was reverse transcribed into cDNA using iScript Reverse Transcription Supermix for RT-Qpcr (Biorad, Hercules, CA, USA). qRT-PCR for selected genes was performed in ABI Vii7 Sequence detection system (PE Applied Biosystems, Foster City, CA, USA). Reactions were carried out in 384-well optical plates containing 25 ng RNA/well and SsoAdvanced™ Universal SYBR^®^ Green Supermix as fluorescent reporter (Biorad, Hercules, CA, USA). Specific primers were designed with Primer3 software [78] using the gene sequences obtained from Affymetrix transcript IDs and obtained from Millipore Sigma (St. Louis, MO, USA). The sequences of the primers used are listed in the Supplement, Table S4. The PCR amplification parameters were initial denaturation step at 95 °C for 10 min followed by 40 cycles, each at 95 °C for 15 s (melting) and 60 °C for 1 min (annealing and extension). For protein coding genes, gene expression was normalized to glyceraldehyde-3-phosphate dehydrogenase (GAPDH) transcription, and for non-coding genes, gene expression was normalized to small nucleolar RNA 68 (SNORNA68) transcription. Relative gene expression was calculated using the delta-delta comparative threshold cycle (Ct) method and expressed as fold-change compared to wild type (WT) mice fed with control diet (CD). 

### 4.9. Bioinformatic Analysis 

Bioinformatics analysis of differentially expressed genes was performed by two of the study investigators (SN and DM) using multiple software tools. We compared each study group (LDL-R −/− WD, LDL-R−/− CD, and WT WD) to the control (WT CD) in female [18] and male mice [19] as previously reported. For fold-change calculations it was also necessary to input experimental group data and compare it to control group data. This information is required by the microarray software (Affymetrix Transcriptome Analysis Console, version 3.1.0.5, Thermo Fisher Scientific, Santa Clara, CA, USA) used in the project.

We first performed bioinformatic analyses of differentially expressed genes with the goal to compare male versus female response to lipotoxicity regardless of the type of lipid stress. This lipid stress could be from high fat diet or/and from genetic variability that can result in high lipid levels in the LDL-R−/− mice. We then performed bioinformatic analysis to assess sex differences in response to different types of lipotoxicity, (i.e., the Western-type diet or/and genetic hyper lipidemia in the LDL-R −/− phenotype). 

The Principal Component Analysis (PCA) plot of identified differentially expressed genes (DEG) was obtained through MetaboAnalyst, Ste Anne de Bellevue, QC, Canada [79,80]. Gene ontology of DEG was done using David bioinformatics database, Frederick, MD, USA [81,82,83]. Canonical pathway analysis was conducted using GeneTrial2 online database, Saarbrücken, Saarland, Germany [84,85] and Metacore software package, Clarivate, Philadelphia, PA, USA [86] to identify significantly over represented pathways. Enrichment statistics were calculated for these data sets assuming a hypergeometric distribution to identify significantly overrepresented pathways. The enriched pathways obtained in our previous step were used to build a network of pathways; two pathways were considered interconnected if at least one of the genes or hits involved in them were common to both. Networks were constructed and visualized using Cytoscape software (version 3.7.1), Ann Arbor, MI, USA [87,88]. Data preparation was performed with the use of several R packages, GitHub, San Francisco, CA, USA included splitstackshape [89], data.table [90], dplyr [91,92] and string [93,94]. Pathway networks were built separately for pathways enriched in each omic layer and pathways obtained from a global pathway enrichment analysis, considering all omic layers components together. To obtain the 6 pathways with the highest degree (number of connections of one node to other nodes), the Cytoscape Network Analyzer application, San Diego, CA, USA was used [95].

Transcription factor analyses were performed using Metacore™, Clarivate, Philadelphia, PA, USA [86]. Hierarchical clustering and heat map representations of differentially expressed genes (DEG) and non-coding RNAs in females and males were performed using PermutMatrix software, Montpellier, France [96,97]. Venn diagrams were generated using Venny, Madrid, Spain [98] and InteractiVenn, São Carlos SP, Brazil [99].Network analysis of interactions between functional groups was identified using Metascape, La Jolla, CA, USA [100,101] and obtained network was visualized using Cytoscape platform, Seattle, WA, USA [87,102]. 

### 4.10. Statistical Methods

For microarray, two-way ANOVA (Affymetrix Transcriptome Analysis Console software, Santa Clara, CA, USA) was used for statistical analysis of microvessel transcriptomes of WD fed WT mice, CD fed LDL-R −/− mice, and WD fed LDL-R −/− mice, each compared to CD fed WT mice. All genes from microarray with *p* < 0.05 and ± 2.0-fold change were considered as differentially expressed. Mean body weight and plasma lipid levels of all 4 diet/genotype groups (CD fed WT, WD fed WT, CD fed LDL-R −/−, WD fed LDL-R −/−) of male and female mice were expressed as means ± standard error of the mean (SEM), and significance determined at *p* ≤ 0.05 using unpaired student’s t-tests (GraphPad software, La Jolla, CA, USA). qRT-PCR determined gene expression in hippocampal microvessels of experimental mice, compared to CD fed WT mice, was expressed as log2-fold change, and statistical significance determined by unpaired student’s *t-*tests (GraphPad software, La Jolla, CA, USA). Y maze cognitive function data was analyzed by unpaired student’s *t*-test (GraphPad software, La Jolla, CA, USA) and expressed as means ± SEM.

## 5. Conclusions and Relevance

Our bioinformatic analyses of differential gene expression in hippocampal microvessels of male and female mice following lipid stress by chronic consumption of the WD demonstrated that female mice had a significantly greater number of DEGs when compared to males. The sex and gender analysis revealed that in females this differential gene expression led to marked sex differences and sex-specificity for several key cellular sequelae that included: (1) the number and pattern of differential gene expression and hierarchical gene clustering, that in turn correlated with differential expression of antioxidant and neuroplasticity processes; (2) the number and functional complexity of cellular pathways and pathway networks targeted by the DEGs in females that related to pathways for regulation of angiogenesis, endothelial cell proliferation and migration, and cellular lipid utilization as well as pathway networks associated with protection against disruption of RNA and oxidative stress-induced DNA injury; and (3) pre and post transcriptional regulation of gene expression by signaling associated with promotion of endothelial cell proliferation, VEGF signaling and angiogenesis, and protection from stress-associated cell death. In contrast, differential expression of male specific transcription factors resulted in cellular signaling that may promote vascular injury by activation of stress induced apoptosis, neurodegeneration, and neuroinflammation.

**Figure 10:**
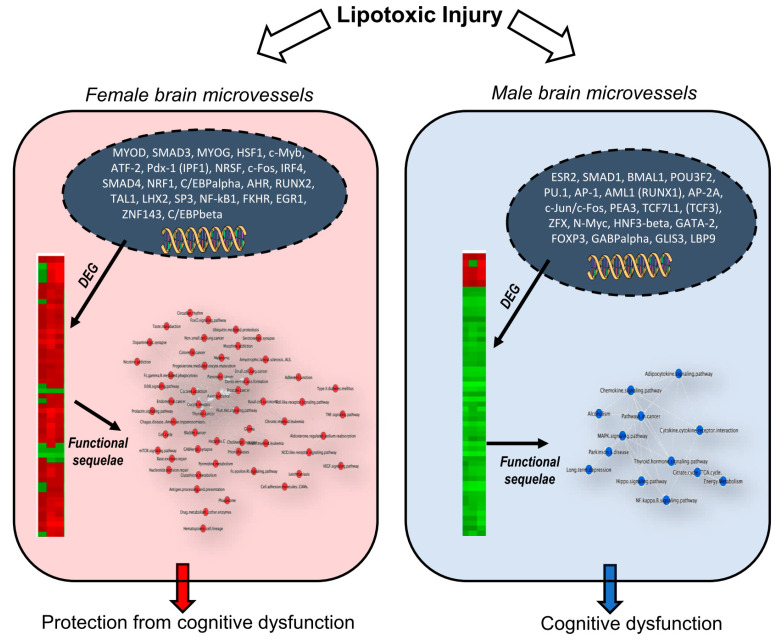
Conceptual Summary of Sex Differences in Molecular Mechanisms of Brain Microvascular Lipotoxicity. Differential expression of transcription factors and genes (DEG) have cellular functional sequelae that result in relative protection from cognitive dysfunction in females compared to males following lipid injury.

The sex differences in the molecular mechanisms of brain microvascular lipotoxicity that we observed in this study, and how they may contribute to protection against neurodegeneration and cognitive dysfunction in females compared to males, is conceptualized in Figure 10. The sex specificity of neuroprotection in females that our studies help to elucidate may help explain some of the epidemiologic differences in Alzheimer’s dementia (AD) between males and females, including the more sustained verbal memory advantage in early stages of AD in women, and help to provide a molecular understanding of the greater cognitive reserve in females compared to males [103]. Given that some AD risk factors are modifiable, such as heart healthy lifestyles and hyperlipidemia, understanding the extent to which sex differences contribute to differential risk for AD and cognitive dysfunction may present important opportunities for new therapeutics. Up to now, the relative lack of consideration of sex and gender in AD has impeded progress in the detection, treatment, and care across the clinical spectrum. Yet, greater attention to sex and gender differences has the potential to improve outcomes for both sexes.

## Supplementary Materials

The following are available online at https://www.mdpi.com/1422-0067/21/21/8146/s1, Figure S1: Mean body weight of wild type (WT) and LDL-R −/− male and female mice pre- and post-feeding with the control (CD) and western (WD) diets, Figure S2: Gene expression by qRT-PCR of genes identified by microarray analysis in male mice hippocampal microvessels, Figure S3: Gene expression by qRT-PCR of genes identified by microarray analysis in female mice hippocampal microvessels, Figure S4. Sex differences in differentially expressed transcription factors in hippocampal microvessels, Figure S5. Sex differences in differentially expressed noncoding RNAs (ncRNAs), Table S1: Plasma lipid levels of wildtype (WT) and LDL-R −/− female and male mice fed with control (CD) and western (WD) diet, Table S2: Plasma Glucose and Insulin levels of wildtype (WT) and LDL-R −/− female and male mice fed with control (CD) and Western (WD) diet, Table S3. List of known differentially expressed genes common between female and male mice shown in Figure 5, Table S4. Primer sequences for genes tested by qRT-PCR were prepared by Primer3 software using Affymetrix transcript ID sequences.
