# Supplementary material for: Sex-Dependent Molecular Mechanisms of Lipotoxic Injury in Brain Microvasculature: Implications for Dementia
Source: Int J Mol Sci. 2020 Oct 31;21(21):8146. doi: 10.3390/ijms21218146 (PMC7663125; doi:10.3390/ijms21218146)
Supplement: Supplementary file 1 [file ijms-21-08146-s001.pdf]

Supplement Figure S1

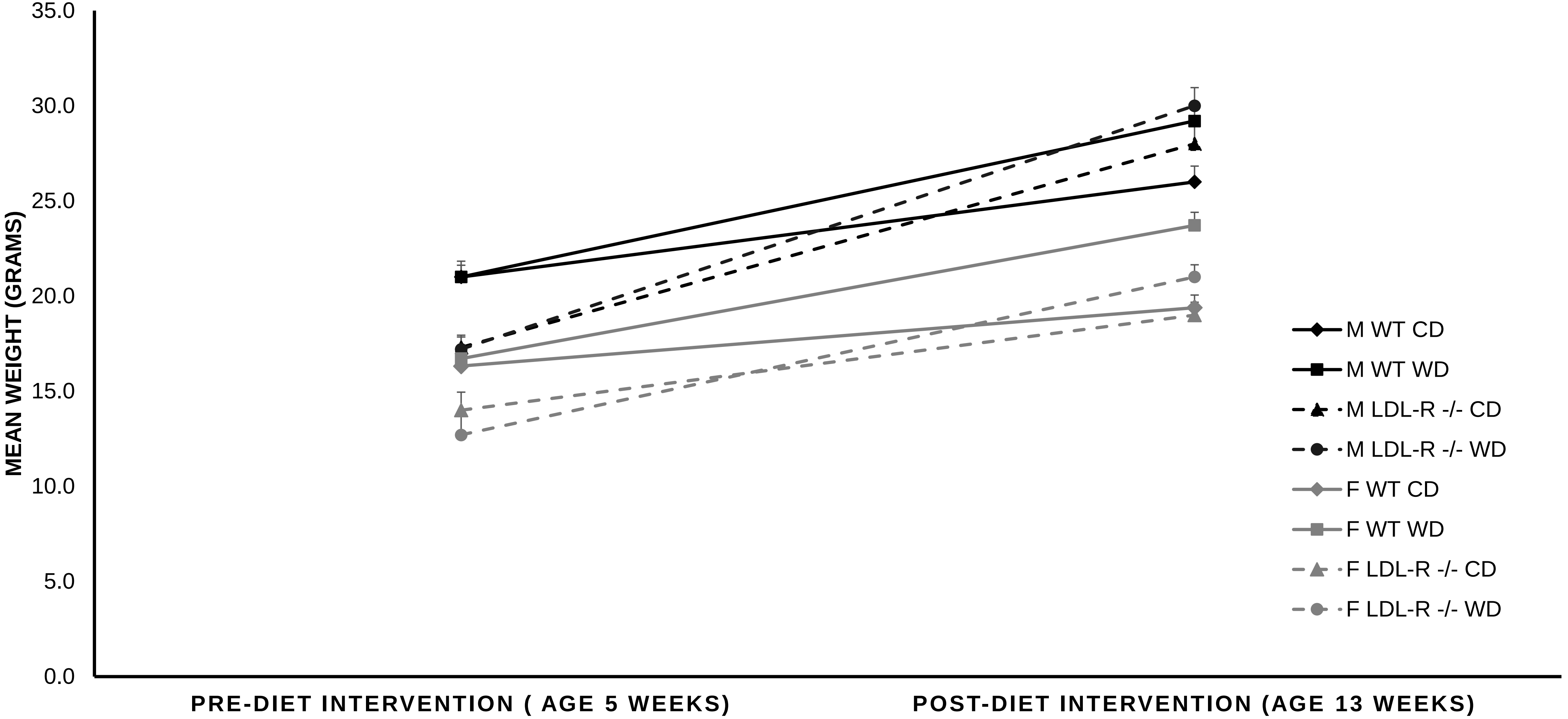

Supplement Figure S1: Mean body weight of wild type (WT) and LDL-R -/- male and female mice pre- and post-feeding with the control (CD) and western (WD) diets. Line graph of mean weight in grams for male (black lines) and female (grey lines) wild type (WT) (solid lines) and LDL-R -/- (dashed lines) mice pre-diet intervention (at age 5 weeks) and 8 weeks post-diet intervention (at age 13 weeks) for the control (CD) and western (WD) diets (n = 7 mice/experimental group).

Supplement Figure S2

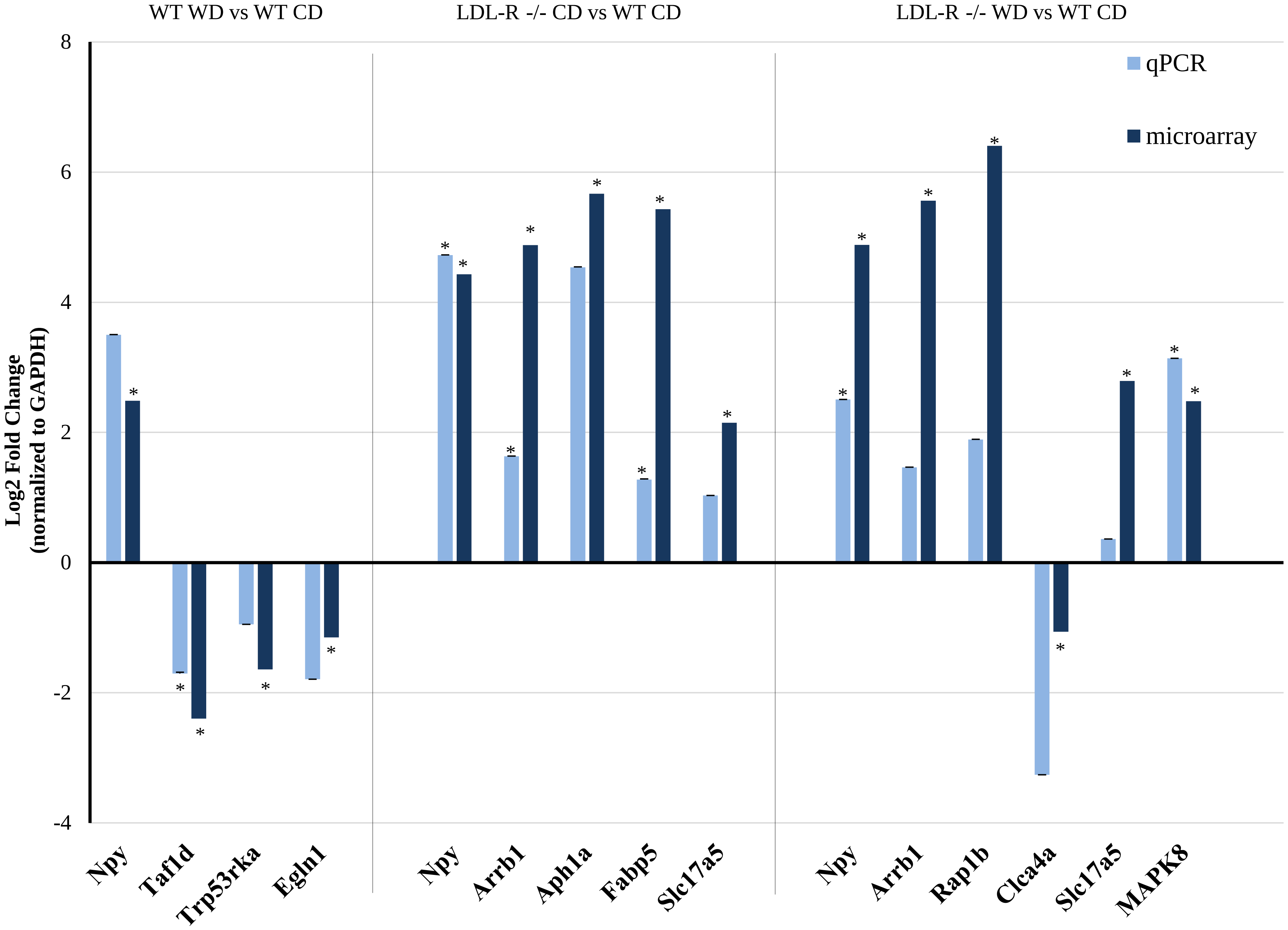

Supplement Figure S2: Gene expression by qRT-PCR of genes identified by microarray analysis in male mice hippocampal microvessels. Eleven protein coding genes (Npy, Taf1d, Trp53rka, Egln1, Arrb1, Aph1a, Fabp5, Slc17a5, Rap1b, Clca4a, MAPK8) were tested by qRT-PCR (n = 7 mice/experimental group) in hippocampal microvessels isolated from wild type (WT) and LDL-R - /- male mice fed with control diet (CD) and western diet (WD) and showed the same trend in gene expression as microarray (n=100 microvessels/mice/experimental group). Protein coding gene expression was normalized to glyceraldehyde-3-phosphate dehydrogenase (GAPDH). Expression levels were expressed as log2 fold change (*$p \leq 0.05$ for WT WD, LDL-R -/- CD, and LDL-R -/- WD when compared to WT CD).

Supplement Figure S3

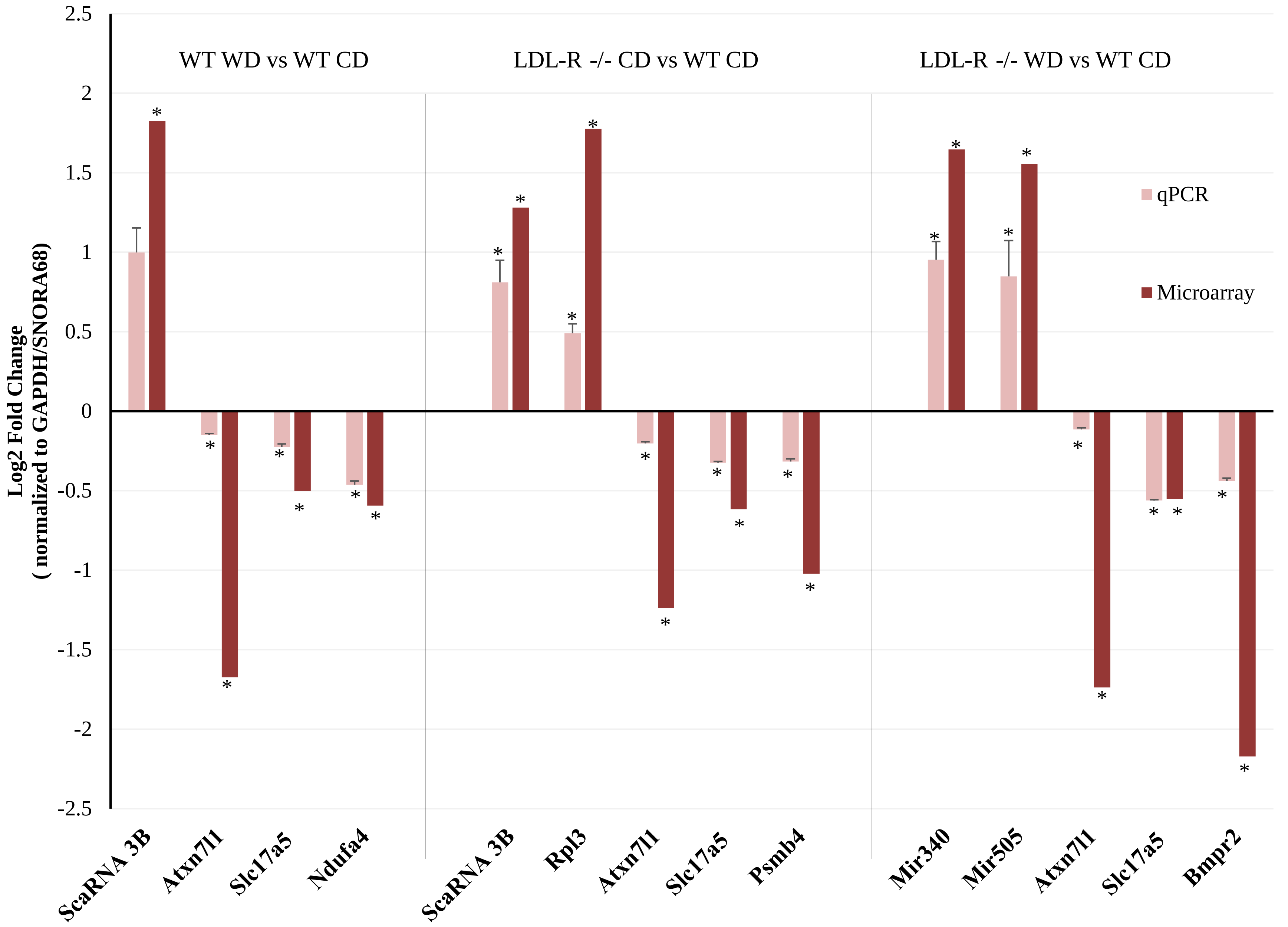

Supplement Figure S3: Gene expression by qRT-PCR of genes identified by microarray analysis in female mice hippocampal microvessels. Six protein coding genes (Atxn7l1, Slc17a5, Ndufa4, Rpl3, Psmb4 and Bmpr2) and 3 non-coding genes (ScaRNA3B, Mir340and Mir505) were tested by qRT-PCR (n = 7 mice/experimental group) in hippocampal microvessels isolated from wild type (WT) and LDL-R -/- female mice fed with control diet (CD) and western diet (WD) and showed the same trend in gene expression as microarray (n=100 microvessels/mice/experimental group). Protein coding gene expression was normalized to glyceraldehyde-3-phosphate dehydrogenase (GAPDH), and non-coding gene expression was normalized to small nucleolar RNA 68 (SNORNA68). Expression levels were expressed as log2 fold change (*$p \leq 0.05$ for WT WD, LDL-R -/- CD, and LDL-R -/- WD when compared to WT CD).

Supplement Figure S4

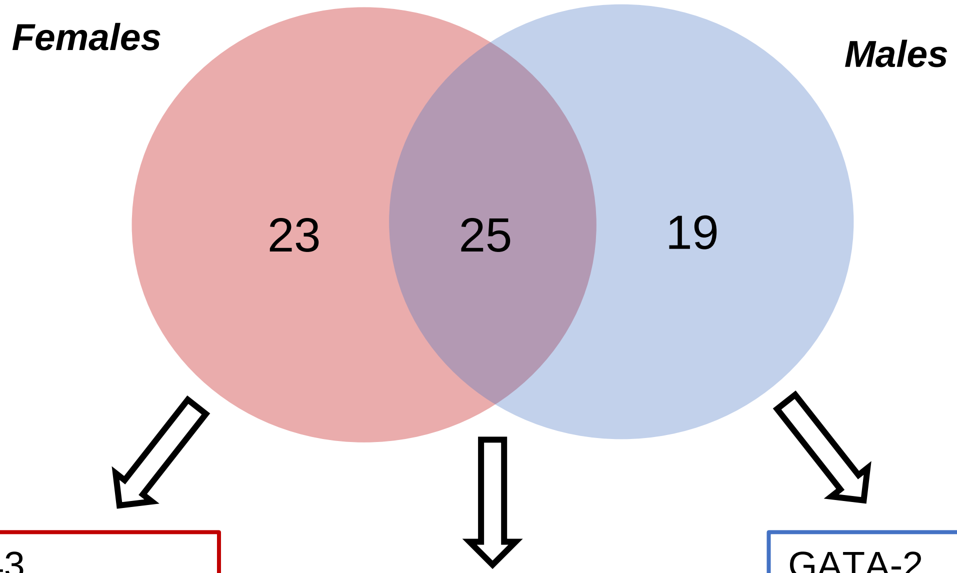

ZNF143
C/EBPbeta
RUNX2
TAL1
MYOD
SMAD3
MYOG
HSF1
c-Myb
ATF-2
Pdx-1 (IPF1)
NRSF
c-Fos
LHX2
SP3
IRF4
NF-kB1 (p50)
FKHR
EGR1
SMAD4
NRF1
C/EBPalpha
AHR

ETS1
Oct-3/4
c-Myc
E2F1
CREB1
YY1
SP1
SOX2
HNF4-alpha
SRF
PPAR-gamma
HIF1A
KLF4
ESR1 (nuclear)
Androgen receptor
NANOG
GATA-1
E2F4
p53
RelA (p65 NF-kB subunit)
GATA-3
STAT3
GCR
c-Jun
TCF7L2 (TCF4)

GATA-2
FOXP3
GABP alpha
ESR2
SMAD1
BMAL1
POU3F2 (BRN2)
PU.1
AP-1
AML1 (RUNX1)
GLIS3
AP-2A
LBP9
c-Jun/c-Fos
PEA3
ZFX
N-Myc
HNF3-beta
TCF7L1 (TCF3)

Supplement Figure S4: Sex differences in differentially expressed transcription factors in hippocampal microvessels. Venn diagram comparing the transcription factors (TFs) between females and males shows 23 female specific TFs (red), 19 male specific TFs (blue) and 25 TFs in common (purple) for all diet/genotype groups (n=100 microvessels/mice/experimental group).

Supplement Figure S5

1700024B18Rik
1700027H10Rik
1700045H11Rik
1700047A11Rik
1700066N21Rik
1700110C19Rik
1700113A16Rik
2310069G16Rik
4632428C04Rik
4930405O22Rik
4930444M15Rik
4930455F16Rik
4930488L21Rik
4930522O17Rik
4930565D16Rik
4930568E12Rik
4933424G05Rik
4933432I03Rik
4933433G08Rik
5330413P13Rik
5430434I15Rik
6330415B21Rik
9430083A17Rik
9530082P21Rik
A930019D19Rik
A930024E05Rik
AF357355
AI314278
AI504432
Arhgap33os
AU022754
B230312C02Rik
C130080G10Rik
C530044C16Rik
D130017N08Rik
D5Ertd605e
Ftx
Gldnos
Gm10007
Gm10010
Gm10390
Gm10619
Gm12637
Gm13003
Gm13411
Gm13790
Gm13985
Gm14061
Gm14254
Gm14684
Gm15409
Gm15413
Gm15556
Gm15590
Gm16295
Gm16793
Gm19784
Gm22
Gm22144
Gm22269
Gm22289/
(Snord116l12)
Gm22323
Gm22378
Gm22485
Gm22504
Gm22531
Gm25860
Gm25945

Gm22546
Gm22757
Gm22759
Gm22774
Gm22840
Gm22883
Gm22932
Gm22935
Gm22940
Gm23000
Gm23031
Gm23119
Gm23121
Gm23129
Gm23134
Gm23136
Gm23199
Gm23527
Gm23534
Gm23722
Gm23734
Gm23931
Gm23970
Gm24013
Gm24096
Gm24098
Gm24127
Gm24252
Gm24284
Gm24313
Gm24327
Gm24328
Gm24336
Gm24400
Gm24429
Gm24449
Gm24504
Gm24524
Gm24581
Gm24627
Gm24648
Gm24668
Gm24678
Gm24682
Gm24766
Gm24770
Gm24771
Gm24813
Gm24844
Gm24878
Gm24916
Gm25068
Gm25125
Gm25371
Gm25381
Gm25401
Gm25410
Gm25443
Gm25526
Gm25559
Gm25607
Gm25715
Gm25720
Gm25777
Gm25817
Gm25856
Gm25982
Gm25992
Gm26047

Gm26070
Gm26154
Gm26272
Gm26387
Gm26593
Gm26643
Gm26656
Gm26675
Gm28890
Gm5144
Gm6410
Hoxaas3
Lincred1
Mhrt
Mir1192
Mir1198
Mir124-1
Mir1938
Mir1954
Mir1955
Mir196a-1
Mir210
Mir21a
Mir28b
Mir30d
Mir343
Mir344d-2
Mir34c
Mir375
Mir377
Mir378d
Mir382
Mir466d
Mir466h
Mir466j
Mir466n
Mir486
Mir5097
Mir5109
Mir574
Mir6418
Mir669n
Mir673
Mir678
Mir687
Mir692-1
Mir692-2
Mir6990
Mir703
Mir761
Mir882
Mir9-2
Pcsk2os2
Plet1os
Rnu3a
ScaRNA15
Snhg7
Snhg7os
Snora17
Snora5c
Snord107
Snord14a
Snord53
Snord66
Snord72
Snord88c
Snord95
Sp3os
Zfp91Cntf

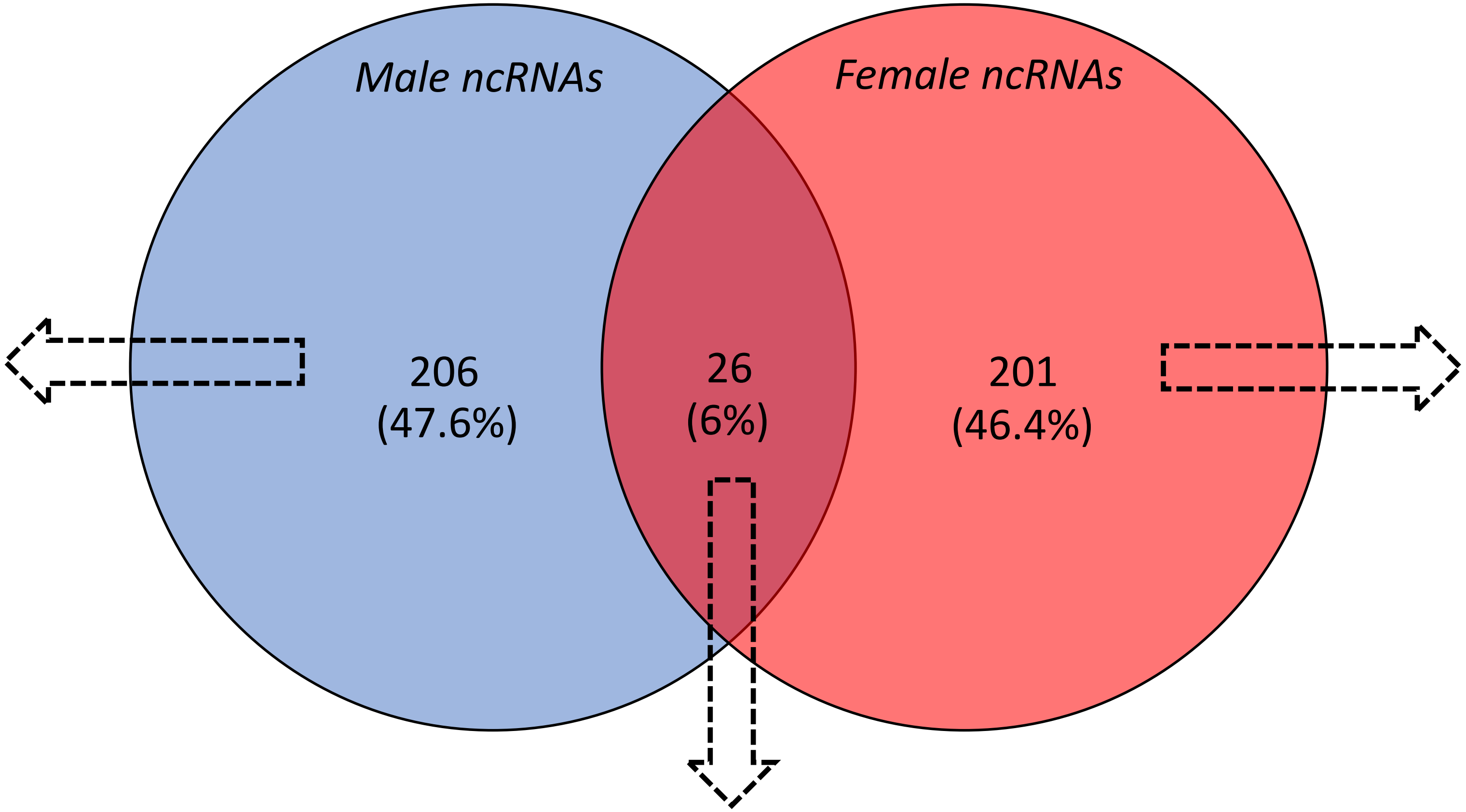

2500002B13Rik
4921534H16Rik
9330102E08Rik
AF357425
Chn1os3
D4Ertd617e
Gm12603
Gm23123
Gm23456
Gm23546
Gm25092
Gm25376
Gm25432
Gm25635

Gm25788
Gm26148
Mir1188
Mir1199
Mir1912
Mir204
Mir466f-4
Mir679
Mir692-3
Snora23
Snord16a
Snord61

1110038B12Rik
1500015A07Rik
1600020E01Rik
1700121N20Rik
2900097C17Rik
4921531P14Rik
4930413F20Rik
4930429F11Rik
4930431F12Rik
4930567H12Rik
4930594O21Rik
4933406C10Rik
4933406D12Rik
4933431E20Rik
5031425E22Rik
5033428I22Rik
7420700N18Rik
9330151L19Rik
9330179D12Rik
A230057D06Rik
A630075F10Rik
AF357428
AU016765
AW495222
Bvht
Cahm
Cep83os
DQ267100
DQ267101
DQ267102
E330033B04Rik
Gm10069
Gm10754
Gm10941
Gm12238
Gm12298
Gm12359
Gm13031
Gm13483
Gm15991
Gm17750
Gm1965
Gm20754
Gm22039
Gm22131
Gm22173
Gm22188
Gm22205
Gm22271
Gm22304
Gm22497
Gm22763
Gm22795
Gm22858
Gm22882
Gm22900
Gm22957
Gm22962
Gm23266
Gm23300
Gm23301
Gm23320
Gm23321
Gm23443
Gm23508
Gm23613

Gm23644
Gm23745
Gm23746
Gm23826
Gm23951
Gm23965
Gm23979
Gm24068
Gm24095
Gm24154
Gm24175
Gm24212
Gm24241
Gm24339
Gm24411
Gm24519
Gm24556
Gm24620
Gm24665
Gm24670
Gm24696
Gm24706
Gm24727
Gm24900
Gm24942
Gm24987
Gm24988
Gm25053
Gm25093
Gm25128
Gm25224
Gm25274
Gm25342
Gm25357
Gm25396
Gm25402
Gm25406
Gm25434
Gm25466
Gm25519
Gm25604
Gm25615
Gm25617
Gm25683
Gm25744
Gm25759
Gm25906
Gm25967
Gm25973
Gm26173
Gm26202
Gm26225
Gm26236
Gm26265
Gm26286
Gm26347
Gm26358
Gm26423
Gm3428
Gm35553
Lyzl4os
Mir1193
Mir1291
Mir15a
Mir181a-2
Mir181c

Mir1843a
Mir1898
Mir1905
Mir191
Mir1941
Mir1943
Mir199a-1
Mir218-1
Mir23b
Mir28c
Mir290a
Mir300
Mir3058
Mir3061
Mir3069
Mir3075
Mir3082
Mir3091
Mir3095
Mir3097
Mir30e
Mir3109
Mir329
Mir337
Mir340
Mir344i
Mir346
Mir362
Mir376b
Mir449b
Mir485
Mir487b
Mir505
Mir5099
Mir5120
Mir5125
Mir539
Mir668
Mir669a-3
Mir669f
Mir680-1
Mir690
Mir694
Mir695
Mir700
Mir8112
Nell1os
Rian
Scarna13
Scarna2
Scarna3b
Snhg14
Snhg17
Snora21
Snora30
Snora34
Snora44
Snord104
Snord11
Snord116
Snord17
Snord34
Snord42b
Snord55
Snord64
Snord65
Snord73a
Snord87
Ttc39aos1

Supplement Figure S5. Sex differences in differentially expressed noncoding RNAs (ncRNAs). Venn diagram of differentially expressed non-coding RNAs (ncRNAs) showing 47.6 % male specific ncRNAs (blue), 46.4% female specific ncRNAs (red) and 6% ncRNAs in common between males and females for all diet/genotype groups (n=100 microvessels/mice/experimental group): C57BL/6J (WT) mice fed western diet (WD), LDL-R -/- mice fed control diet (CD) and LDL-R - /- mice fed WD, when compared to WT mice fed CD.

Table S1: Plasma lipid levels of wildtype (WT) and LDL-R -/- female and male mice fed with control (CD) and western (WD) diet.

Plasma Lipids	WT-CD		WT-WD		LDL-R -/- CD		LDL-R -/- WD	
	Female	Male	Female	Male	Female	Male	Female	Male
	Mean±SEM							
TC (mg/dL)	73.9±10.0	89.3±1.6	119.8±7.1*	252.8±21.5	225.7±24.0	285.6±41.1	1259.6±59.5	1151.8±38.1
HDL (mg/dL)	57.1±8.4	77.6±1.5	95.1±5.8*	201.0±16.7	87.5±11.7	116.4±15.8	113.5±14.3	116.2±16.5
LDL (mg/dL)	0.93±2.0	6.1±0.8	14.5±0.6*	44.6±7.2	113.0±17.2	134.9±39.1	1038.6±39.7	983.1±17.0

** p <0.05 for females compared to males for each diet-genotype group.*

N=3 per diet/genotype group.

Table S2. Plasma Glucose and Insulin levels of wildtype (WT) and LDL-R -/- female and male mice fed with control (CD) and Western (WD) diet.

	WT-CD		WT-WD		LDL-R -/- CD		LDL-R -/- WD	
	Female	Male	Female	Male	Female	Male	Female	Male
	Mean±SEM							
Glucose (mg/dL)	172.1±50.4	292.1±0.1	430.4± 34.6	494.3± 2.7	303.0± 23.1	345.1± 14.1	424.1± 27.9	360.0±21.5
Insulin (mg/dL)	102.9±11.7*	232.0±9.9	132.6± 46.8*	918.1± 57.7	249.6± 34.3	349.4± 33.6	179.0±54.6*	462.7± 80.4

** p <0.05 for females compared to males for each diet-genotype group.*

N=3 per diet/genotype group.

Table S3: List of known differentially expressed genes common between female and male mice shown in Figure 5.

Gene symbol	Females	Males		Gene symbol	Females	Males
	Fold change				*Fold change*	
Gm2399	129.21	112.35		Caskin1	3.03	3.08
LOC105245453	129.21	112.35		Plekho2	3.01	3.73
Syne1	73.27	73.78		Colgalt2	2.96	2.38
Chtf8	62.9	29.38		Maneal	2.94	7.82
Gm24627	58.13	2.08		Fstl5	2.89	2.03
Mir1912	37.22	2.11		Gm9861	2.88	2.17
Cops6	28.22	26.39		Litaf	2.88	2.17
Snhg7	27.77	11.93		Igsf5	2.85	2.02
Snora17	27.77	11.93		Gm36490	2.82	55.42
Snord16a	26.26	3.73		Ttc34	2.82	2.15
Mir1188	25.82	18.6		Ppfibp1	2.81	4.03
Lgals1	24.45	81.38		Rpap1	2.81	2.46
Gm9396	23	10.09		Agk	2.8	4.1
Gm24009	22.97	63.73		Nr2f6	2.76	3.29
Gm24217	22.01	24.57		Arrb1	2.74	29.42
Kctd13	21.87	25.72		Sec23ip	2.74	2.15
Gm25376	21.03	4.27		Nacc2	2.71	8.37
mt-Tv	16.11	14.78		Snrnp70	2.71	15.8
Mir679	14.7	113.69		B4galnt4	2.67	11.32
Psip1	14.49	2.45		Ccm2	2.57	2.85
Snora23	14.28	8.96		Tmem19	2.56	2.62
Spag7	13.94	2.18		Gm15446	2.55	2.73
Cep83os	11.85	49.06		Pdia6	2.52	4.16
Gm25559	11.7	11.52		Prrt4	2.51	4.13
Taf1d	10.62	-5.27		Cdk5rap1	2.5	2.83
LOC102640399	10.39	16.41		Tmem121	2.49	7.99
Gm12396	9.99	11.37		9430024E24Rik	2.46	2.2
Gm6644	9.89	5.82		Mfsd5	2.45	9.35
D4Ertd617e	9.82	12.6		Paip1	2.45	2.23
Mir204	9.26	4.11		Cspp1	2.44	2.85
Ppp1r1b	9.19	3.87		Slc43a2	2.44	2.21
Aph1a	9.08	50.78		Gbp3	2.42	4.27
Zfp938	8.72	29.1		Sgf29	2.41	5.26
Rps11-ps1	8.5	9.77		Gdpd3	2.4	2.13
Ifitm3	8.43	15.04		Gm7609	2.39	2.43
Fars2	7.94	16.11		2210011C24Rik	2.34	3.76
Gm25788	7.48	29.72		4921534H16Rik	2.34	2.24
Epas1	7.23	20.12		Crocc	2.34	2.99
Rps15a-ps5	7.21	10.93		Mir1199	2.34	3.76
Dap	6.88	10.38		Chn1os3	2.28	3.42
Raver2	6.82	2.75		1110051M20Rik	2.25	4.04
Bex1	6.61	22.92		Ermp1	2.25	2.38
Trdv2-2	6.48	14.18		Acta1	2.24	6.51
Ankhd1	6.47	14.44		Pdlim1	2.24	5.99
Gm23456	6.44	5.32		Upp1	2.24	2.04
Txnl4b	6.37	11.13		Gm12603	2.23	2.59
Parp2	5.85	5.91		Ror1	2.22	3.07
Egln1	5.69	-2.22		Mfsd11	2.19	5.07
3110039I08Rik	5.63	29.55		Ripk3	2.19	2.03
Zbtb39	5.52	3.23		Gm20257	2.18	21.53
Gm26148	5.5	4.58		Mterf1a	2.17	2.46
Cadm4	5.41	10.93		Gm16299	2.15	2.11
Vmn2r33	5.29	2.78		Smok3a	2.15	2.07
Pglyrp1	5.12	5.71		Smok3b	2.15	2.07
Zfp128	4.62	2.47		Gdap1	2.12	5.51
Tcrg-V3	4.58	2.13		Nova2	2.11	4.23
Araf	4.39	10.1		Hpn	2.1	2.35
Gm38487	4.38	7.29		Fam159b	2.06	2.81
Stxbp6	4.38	7.29		Gm12665	2.03	2.05
Pdcd5	4.34	2.34		LOC102636777	2.03	2.05
Tubb2a	4.24	5.54		2500002B13Rik	2.01	2.07
LOC102637947	4.23	2.9		Gm26151	2.01	2.34
LOC101056074	4.22	3.27		Tk2	2.01	5.98
Cdc42se2	4.15	10.28		Sft2d1	-2.29	9.35
Trim43c	3.94	4		Idh3g	-2.4	23.71
Gm15710	3.87	7.57		Slc25a44	-2.44	4.94
Mcee	3.77	6.8		Cycs	-2.49	35.27
Dtx1	3.64	2.67		Zfr	-2.58	3.54
Fgf1	3.64	7.24		Gdpd1	-2.81	2.05
Lrp3	3.63	5.89		Gm25432	-3.3	3.18
Gm4609	3.54	4.87		Lamp1	-3.4	20.45
Gm25092	3.49	3.89		Exoc3	-3.42	12.68
9330102E08Rik	3.46	2.51		Med13l	-3.67	5.35
Gm24878	3.45	9.74		Fntb	-3.7	2.07
Pold1	3.4	3.09		Gm24766	-4.04	10.11
Gm23123	3.34	9.93		Gm25817	-4.04	10.11
Nmnat2	3.27	4.8		Gm1673	-5.88	12.92
Tmem254a	3.23	8.09		Ftl1	-6.4	20.29
Tmem254b	3.23	8.09		Gm15590	-6.4	20.29
Tmem254c	3.23	8.09		Gm22774	-6.4	20.29
Gm26675	3.21	4.4		Mir692-3	-6.4	17.54
Klf1	3.18	2.2		Itm2b	-7.15	11.09
Tor2a	3.16	2.38		Ttc9b	-7.24	13.42
Gm10715	3.13	5.09		Gm25635	-7.41	90.8
Dbndd2	3.12	3.06		Npy	-8	5.61
Gm3086	3.07	4.83		Psma2	-10.57	2.11
Akr1e1	3.06	2.75		Gm16089	-10.59	21.75
Rnf169	3.06	15.49		Fabp5	-28.82	49.88
				Snord61	-33.17	-39.34

Table S4: Primer sequences for genes tested by qRT-PCR were prepared by Primer3 software using Affymetrix transcript ID sequences.

Gene	Primer Sequence (5'-3')
Gapdh	Sense-GCAACAGGGTGGTGGACCT
	Antisense GGATAGGGCCTCTCTTGCTCA
Snora68	Sense-TAGTGGTGCACACAGCAAAG
	Antisense -AGAGCCTACTGATTCACTCTGG
Npy	Sense- CCGGTGGATCTCTTCTCTCA
	Antisense- CCCCATTCGCTTGTTACCTA
Taf1d	Sense- GCATGGTATCTGCACTCAGC
	Antisense- ATGCACAAAGCCAAGAAACC
Trp53rka	Sense- CCTACGTGGGTGTCTGGAGT
	Antisense- ACCACACTGGAGTCCTTTGG
Egln1	Sense- TACAGGATAAACGGCCGAAC
	Antisense- CGCATCTTCCATCTCCATTT
Arrb1	Sense- TCCTGGCACAGAGACACTTG
	Antisense- AATTCATTCCCAAGGTGCAG
Aph1a	Sense- TATGGCCTCCTGATTTTTGG
	Antisense- GATGCTAAGCCCTCATCTGC
Fabp5	Sense- TGCAACAAACAGCTTCACTTC
	Antisense- TCCTGGGTAAACCAAGTTTGA
Slc17a5	Sense- CCCCTGTTACAGCCACTGTT
	Antisense- TTTCCCTCGTGCTTGTCTCT
Rap1b	Sense- GCTCTGAGCCAGGTCTGAAG
	Antisense- CACCACAGGAAAGTCCGTTT
Clca4a	Sense- CATCCACTTCACCCCTGACT
	Antisense- AAATACTCCCCAGCGAAGGT
Mapk8	Sense- TTTTGCTGTGAAACTTTTGATTATCA
	Antisense- AACTTAACATGTGGTGCAATTTCTGT
ScaRNA3b	Sense-GAAAATGCCTTTGTTTGCAG
	Antisense -CGATCAGACTCAGCCAGCTA
Atxn7l1	Sense-GGCGTGGAAGACATTAGGAA
	Antisense-TACTGTGTTGGCGTCTCCTG
Slc17a5	Sense-CCCCTGTTACAGCCACTGTT
	Antisense-TTTCCCTCGTGCTTGTCTCT
Ndufa4	Sense-GGCTGATGTAAGGCCATGTT
	Antisense-TAAAGCTGTGTGCTGCCATC
Rpl3	Sense-GATAATGTGAGACCCTGGAACG
	Antisense-AGAAGGCAGGCAGGCAAG
Psmb4	Sense-TGAAGTGTTCGCTGAGTTGG
	Antisense-TCTAGCTTTTGGGAGGCAGA
Mir340	Sense-CAATTGTACTTGGTGTGATTATAAAGC
	Antisense-AGTAACTGAGACGGATCCCACA
Mir505	Sense -GTGGGGGAGCCAGGAAGTAT
	Antisense -TGATACTCCAGAGAGAAAACCAG
Bmpr2	Sense-AGTTGTGTGACGGAGCAGTG
	Antisense-TCAGCGTTCATAGTGGCATC
